# Cold pressed oils: a systematic review on physicochemical properties, nutritional composition and pharmacological activities

**DOI:** 10.3389/fnut.2026.1747029

**Published:** 2026-07-01

**Authors:** Biswabhusan Dash, Ashirbad Sarangi, Ananya Nayak, Bhabani Shankar Das, Sudipta Jena, Asit Ray, Pratap Chandra Panda, Sanghamitra Nayak, Ambika Sahoo

**Affiliations:** Centre for Biotechnology, School of Pharmaceutical Sciences, Siksha ‘O’ Anusandhan (Deemed to be University), Bhubaneswar, Odisha, India

**Keywords:** antioxidants, carotenoids, cold-pressed oils, phytosterols, polyphenols, polyunsaturated fatty acid

## Abstract

**Background:**

In recent years, there has been increasing interest in novel plant-based oil sources as sustainable alternatives to conventional edible oils. This shift is driven by consumer demand for natural, nutrient-dense, and health-promoting products. Among these, cold-pressed oils have gained prominence as specialty oils. Cold-press extraction is a mechanical, environmental friendly and energy-efficient technique that eliminates the use of heat and solvents, thereby preserving the natural physicochemical and nutritional properties of oils. This method is applied to seeds, kernels, fruits, and peels to obtain oils rich in bioactive compounds.

**Aim of the review:**

This systematic review aims to summarize and critically evaluate the extraction techniques, stability-enhancing strategies, physicochemical characteristics, nutritional composition, and pharmacological activities of various cold-pressed oils. It also highlights current research gaps to guide future investigations.

**Methods:**

Relevant literature was collected from scientific databases, including PubMed, Scopus, Web of Science, Google Scholar, and NCBI, as well as peer-reviewed journals. Additional gray literature, including web articles were also examined to ensure a comprehensive review of available information.

**Results:**

Cold-pressed oils retain significantly higher levels of polyunsaturated fatty acids, antioxidants, vitamins, and other bioactive compounds compared to refined oils due to minimal processing. These constituents contribute to enhanced biological activities, including antioxidant, anti-inflammatory, and anticancer properties. Consequently, cold-pressed oils exhibit improved nutritional quality and potential health benefits. However, their quality and shelf life are strongly influenced by factors, including the source of raw materials, plant genotype, harvest timing, moisture content, storage conditions, pre-processing steps, and extraction parameters. Oils rich in mono-and polyunsaturated fatty acids are particularly susceptible to oxidation, which can compromise stability and health benefits. To address this, various strategies, including the use of natural antioxidants, encapsulation, optimized storage conditions, and advanced extraction technologies, have been proposed to enhance oxidative stability.

**Conclusion:**

Cold-pressed oils offer valuable therapeutic and nutritional benefits due to their rich profile of bioactive compounds. Despite their potential, research gaps remain, in developing integrated approaches to maintain and enhance their stability, nutritional value, and pharmacological properties. Addressing these gaps is crucial for fully understanding and maximizing the health benefits of plant-based, cold-pressed oils.

## 1 Introduction

Cold-pressed oils play a key role in modern nutrition science, functional food research, and natural products research due to their bioactive compounds and minimal processing. Cold-pressed oils are mechanically pressed at temperatures lower than 40–60 °C, thereby preserving thermolabile nutrients and phytochemicals. In contrast, oils processed by refining or solvent extraction undergo high-temperature treatments, including bleaching and deodorization (1). This extraction method aligns with global consumer demand for minimally processed, “clean label” foods and offers a superior nutritional and therapeutic profile compared to traditional oils. As consumer habits shift (toward more preventive measures), cold-pressed oils have garnered both scientific and commercial attention due to their diverse health benefits. The worldwide prevalence of chronic diseases, particularly cardiovascular disease, diabetes, metabolic syndrome, and chronic inflammatory diseases, has prompted a broad range of research on dietary lipids as modulators of physiological activity.

Edible cold-pressed oils from seeds, nuts, and fruits (olive, flaxseed, sesame, coconut, hempseed, avocado, pumpkin seed, groundnut, mustard, pomegranate), composed of polyunsaturated fatty acids (PUFAs), monounsaturated fatty acids (MUFAs), tocopherols, phytosterols, carotenoids, squalene, and other phenolic compounds, are available (2). They serve vital functions in lipid homeostasis, oxidative stress defense, inflammation modulation, and neurological, endocrine, and skin health. On the contrary, processed oils lose 20–80% of the antioxidant constituents during oil refining; thus, a considerable reduction in the amount and functional capacity has been observed (3). Cold-pressed oil composition is closely related to species, cultivar, country and geographic origin, agroclimatic conditions, seed maturity, as well as extraction conditions. Flaxseed oil has a high content of α-linolenic acid (ALA) (up to 52%), which makes it a different oil from olive and avocado oils [oleic acid-rich oils, also high oxidative stability and cardioprotective properties; (4)]. Unique lignans, such as sesamin and sesamolin, which enhance both antioxidant potential and shelf stability, are also found in sesame oil (5). Cold-pressed coconut oil, which contains a large amount of MCFAs (medium chain fatty acids) like lauric acid, can also be regarded favorably since it is used to create high yield, antiseptic, and anti-inflammatory (e.g., quick metabolic oxidation) properties (6). It is this variation in the chemical composition that underpins the unique nutritional and therapeutic profiles for each oil.

Cold-pressed oils also contain a greater percentage of unsaponifiables than refined oils. Such fraction contains compounds such as phytosterols, triterpenes, aliphatic alcohols, and lipid-soluble vitamins, which are largely bioactive molecules, although relatively few to moderate compounds (7). The synergistic relationship among fatty acids, phenolics, carotenoids, sterols, and tocopherols boosts the oils’ antioxidant, anti-inflammatory, and metabolic control functions. Additionally, the phenolic compound oleocanthal in extra-virgin olive oil functions similarly to ibuprofen, inhibiting cyclooxygenase enzymes. Furthermore, γ-tocopherol in pumpkin seed and hempseed oil is also responsible for scavenging reactive nitrogen species (8, 9). There is an emerging line of evidence indicating the potential therapeutic applications of cold-pressed oils across a broad spectrum of biological environments. Strong radical-scavenging activity has been reported for phenolic and flavonoid compounds *in vitro*, while *in vivo* studies have also indicated enhancements in lipid metabolism, glycemic and oxidative biomarkers, as well as inflammatory markers (10). Moreover, they have been found to contribute to cardiovascular health in clinical trials, where results revealed that the reduction of LDL oxidation, increased arterial elasticity, and modification of lipid profiles were observed after dietary inclusion of oils of the family (flaxseed, olive, sunflower, and walnut) (11, 12). These results support the wider framework of dietary lipids as bioactive agents (vital agents rather than just energy sources).

Despite these advantages, cold-pressed oils are susceptible to oxidation because of their high PUFA content. And with oils such as flaxseed, hempseed, walnut and more like this, they have the potential for oxidative damage. Oxidative degradation leads to rancidity, reduced nutrition, and potentially hazardous oxidation products (13). Storage conditions, including light exposure, available oxygen content, temperature, and packaging, are key factors determining the oxidative stability of these oils. New research focuses on natural antioxidant fortification, controlled inert-atmosphere extraction, microencapsulation that preserves antioxidants over time, and the use of amber or UV-protective packaging to achieve longer shelf life (14).

For safety, uniformity, and therapeutic efficacy, it is important to establish suitable extraction processes along with quality control variables. In addition, global market demand has increased the likelihood of adulteration, particularly in high-value oils such as olive, argan, and avocado oils. Data analysis using chromatographic, spectroscopic, DNA-based, and metabolomics approaches is increasingly essential to ensure the purity and safety of consumer products (15). These analytical strategies enhance traceability, validate botanical sources, and enable the detection of blending with lower-cost oils.

Briefly, cold-pressed oils are poised at the interface of nutrition, biochemistry, and therapeutic science. However, their composition, rich in bioactive compounds and combined with a low level of treatment, makes them great alternatives to refined oils for human health. Challenges such as oxidative instability, lack of standardization, and adulteration highlight the importance of ongoing research and regulatory scrutiny. These key studies were complemented by a systematic review that provided an overview of the existing knowledge on the physicochemical characteristics, nutritional composition, bioactive compounds, and pharmacological properties of the main cold-pressed oils. This review highlighted the key research gaps found and provided future research directions.

Although previous reviews have discussed edible oils, this study provides a systematic and integrated analysis of cold-pressed oils by linking extraction technology with physicochemical properties, composition, and reported bioactivities. Unlike earlier works that focus mainly on health benefits, this review emphasizes source-dependent variability and critically evaluates the evidence level of reported effects. It also highlights key research gaps, including the need for standardization and clinical validation, thereby offering clearer directions for future research.

## 2 Methods

### 2.1 Study design and guideline compliance

The systematic review was conducted in accordance with the guidelines established by PRISMA 2020 (Preferred Reporting Items for Systematic Reviews and Meta-Analyses), ensuring transparency, methodological rigor, and reproducibility. The review design encompassed several predefined phases, including the development of a search strategy, study selection, eligibility assessment, data extraction, quality evaluation, and qualitative synthesis.

### 2.2 Search strategy and data sources

A comprehensive literature search was performed across major scientific databases, including PubMed, Scopus, Web of Science, ScienceDirect, and Google Scholar. The search aimed to identify studies that reported the physicochemical characteristics, nutritional composition, and pharmacological activities of cold-pressed oils. The strategy incorporated controlled vocabulary and free-text terms such as “cold-pressed oil,” “cold press extraction,” “physicochemical properties,” “nutritional composition,” “bioactive compounds,” “pharmacological activity,” “antioxidant,” “anti-inflammatory,” and “fatty acid profile.” The search covered records from the database’s inception to November 2025 and included only English-language publications, without geographical restrictions.

### 2.3 Study selection and eligibility criteria

Identified records were exported into reference management software to remove duplicates. Titles and abstracts were screened independently to determine relevance. Studies were eligible if they met the following criteria: (i) Oils obtained exclusively through cold-press extraction; (ii) Evaluation of at least one physicochemical parameter (e.g., peroxide value, acid value, iodine value, saponification value, oxidative stability); (iii) Reported nutritional or compositional attributes (fatty acid profile, tocopherols, phytosterols, carotenoids, polyphenols, minerals, vitamins); (iv) Documented pharmacological or biological activities (antioxidant, anti-inflammatory, antimicrobial, anticancer, hepatoprotective, cardioprotective, metabolic) using *in vitro*, *in vivo*, or *ex vivo* models. Excluded studies consisted of those using solvent-extracted, refined, or heat-treated oils, review articles, conference abstracts, patents, and studies lacking adequate analytical data.

### 2.4 Full-text retrieval and data extraction

Full-text articles meeting the inclusion criteria were retrieved and thoroughly reviewed. Data extraction followed a standardized template, capturing study characteristics (authors, year, geographic origin, and oil type), extraction conditions, analytical methodologies, physicochemical parameters, nutrient composition, bioactive compound concentrations, and pharmacological outcomes. Where necessary, means, ranges, and standardized values were recalculated to allow cross-study comparisons.

### 2.5 Quality assessment and risk of bias evaluation

Methodological quality and risk of bias were assessed using appropriate appraisal tools. The Joanna Briggs Institute (JBI) checklist was applied to experimental and observational studies, while a modified quality assessment tool for laboratory-based *in vitro* studies was used to evaluate methodological clarity, replicates, controls, and analytical validity. Studies were categorized as having low, moderate, or high risk of bias based on predefined scoring cutoffs.

### 2.6 Data synthesis and reporting

Findings were synthesized qualitatively due to heterogeneity across oil types, analytical techniques, and experimental models. When adequate data were available, the physicochemical and nutritional profiles of the oils were compared to determine trends, variations, and influencing factors, including seed type, geographical origin, and processing conditions. Pharmacological activities were grouped into five categories: antioxidant, anti-inflammatory, antimicrobial, metabolic, and organ-protective effects. A meta-analysis was not conducted due to variability in measurement units, analytical methods, and study designs. This methodological approach ensured comprehensive coverage of the available evidence concerning the physicochemical traits, nutritional richness, and biological potential of cold-pressed oils.

To improve clarity and comparability, the included studies were categorized based on oil source (seed, nut, and fruit-derived oils), and their key physicochemical parameters were systematically compared. A structured synthesis of FFA, PV, IV, and SV values across oil types revealed consistent trends linked to fatty acid composition and botanical origin. PUFA-rich oils generally showed higher oxidation indices, whereas MUFA- and SFA-dominant oils exhibited greater stability. Although heterogeneity in analytical methods limited formal meta-analysis, cross-study comparison enabled identification of consistent directional trends and relative effect differences. Furthermore, all interpretations were supported by multiple studies to ensure adequate referencing and strengthen the reliability of the conclusions.

## 3 Results

**Figure 1:**
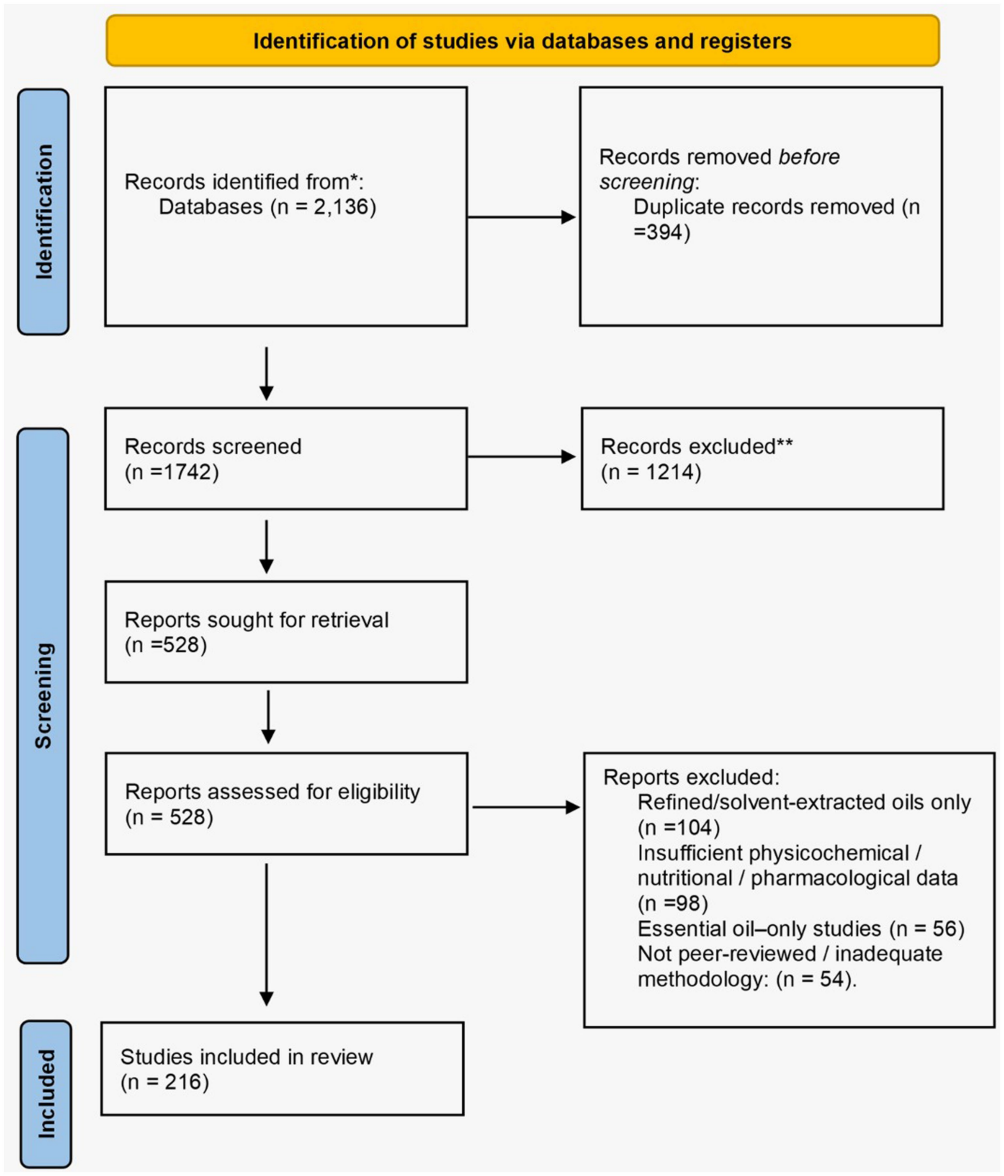
PRISMA flow diagram for literature search.

A total of 2,136 records were retrieved from the database searches and imported for screening. After removing 394 duplicate entries, 1,742 unique records remained for title and abstract screening. Of these, 1,214 were excluded due to clear irrelevance to the review objectives. The remaining 528 reports were thoroughly assessed for eligibility. Following detailed evaluation, 104 studies were excluded for focusing exclusively on refined or solvent-extracted oils, 98 for lacking sufficient physicochemical, nutritional, or pharmacological data, 56 for examining essential oils only, and 54 for inadequate methodology or absence of peer review. Ultimately, 216 studies met all inclusion criteria and were incorporated into the final systematic review. The overall screening and selection process is summarized in the PRISMA flow diagram (Figure 1).

### 3.1 Methods of extracting cold-pressed oil

Cold-pressed oils are prepared from oil-bearing plants that contain over 15 percent oil, seeds, or fruit. Cold pressing is still one of the most traditional natural methods of oil extraction, with safety in its absence by the absence of any chemical solvents considered safe (GRAS) by the U.S. Food and Drug Administration. Oil used to flow up through a wooden pest powered by a bull. Modern methods utilize power-operated cold-press meals, generally hydraulic or screw-type expeller processes made of stainless steel with additional cooling equipment. Screw presses or expellers use a rotating screw to grind and compress oilseed material, pushing the oil out through small holes. This mechanism may result in increased heat due to friction. Production is often run to hold temperatures low enough (of around 50 °C and 122 °F) to preserve manufacturer branding as “cold-pressed” (16). A hydraulic press crushes the raw material sufficiently to allow for oil extraction to occur within a short period. Pre-conditioned steps, including dehulling and milling, are used to prepare the seeds for maximum oil yield (17). The temperature during pressing, seed pretreatment, and moisture content of the raw material significantly influence oil yield and its chemical profile. Moderate seed moisture (6–8%) facilitates better oil release, while excessive moisture can lead to hydrolytic degradation and increased free fatty acid (FFA) levels. Similarly, mild mechanical pretreatment such as light roasting or dehulling may enhance oil recovery but must be carefully optimized to prevent oxidation of delicate fatty acids (18). This process is scientifically simple, economically practicable, and it preserves the properties of the thermolabile bioactive compounds (2, 19).

When compared to solvent extraction, cold pressing typically yields lower oil volumes but higher nutritional quality. Solvent extraction utilizes organic solvents (e.g., hexane) to achieve a high yield, while also eliminating advantageous compounds and introducing residues. In contrast, cold-pressed oils preserve natural antioxidants, pigments, and flavor compounds; thus, they are superior to solvent-extracted oils in wellness applications, albeit with a shorter storage time and lower total production efficiency (20). Cold pressing by-products are quite useful, notably the remaining seed cake. These cakes contain a significant amount of nutrients and can be used as animal feed, fertilizer, or as raw materials to produce other useful products, such as proteins and polysaccharides. This enables a more viable zero-waste or circular economy model (21).

### 3.2 Stages of cold pressing

Cleaning and washing of raw material: The raw materials are cleaned to remove physical impurities, such as dust, dirt, and adhering particulates. Rinsing thereafter cleans residual pesticides as well as surfaces that harbor microbial flora, providing clean, contaminant-free raw material for oil extraction.

Defatting: This process has been shown to improve both oil and meal quality, as the oils are clearer (with a lighter color) and more oxidation-stable. Despite technical restrictions, the oil extract can be used in large-scale industrial practices to eliminate unwanted compounds, such as chlorophylls, trace metals, and pesticides, that are likely to diffuse through the hulls or shells into the oil.

Fragmentation (Crushing): Partial mechanical disruption of the seed structure and hull results in increased oil release through cell rupture, an enlarged oil exudation surface, and reduced internal resistance. The crushed seeds are rapidly examined for pressing and extraction of oil while also being drained to maintain their quality and oxidative stability for cold-pressed oils.

Conditioning: This procedure involves adjusting the moisture content of the raw materials to achieve an optimal humidity level. This promotes the separation of tissue and oil glands, thereby improving oil extraction. Roasting involves heating the pulp to around the temperature of 100 °C (which is also considered) and can also include adjusting the moisture content to get humidity as a bonus (22). Although this method increases oil removal efficiency, it results in a decline in both oil and meal quality. The quality of cold-pressed oils depends on several factors, including the properties of the raw materials (such as purity, uniformity, integrity, and ripeness), harvest timing, and pre-pressing methods (e.g., crop collection, drying, storage, and handling).

Hydraulic press oil extraction was studied, and it has been demonstrated that whole seed roasting before extrusion reduces lipid hydrolysis and influences acid value considerably less than milling seeds. Milling seeds (to minimize the amount or temperature of oils) and conditioning at a temperature of 60–80 °C provided the best oil quality. This resulted in a higher yield, leading to oxidative stability and significantly fewer adverse variations, such as deterioration of taste, increased acid and peroxide values, and a higher pigment content (23).

In screw-type presses, oil can be extracted under mild conditions without changes in fatty acid profiles/oxidation indices, regardless of nozzle diameter (24). However, the extraction efficiency and residual fat in the pomace were lower. Pre-press thermal treatment markedly increases the oil production but also induces color darkening, lipid hydrolysis, and pheophytin accumulation in the product (25).

Oil collection: Oil is extracted from raw materials through mechanical crushing, and then it is collected into a container, leaving it to settle by sedimentation. The clarified oil is further filtered and filed away before being packaged in PET or glass bottles. Oil should be stored in lightproof, airtight, and moisture-resistant containers under cool conditions to extend its shelf life for several months. Proper handling and storage preserve the fatty acid composition and maintain both the primary and secondary oxidation indices, thereby ensuring the oxidative stability of the cold-pressed oils (220).

### 3.3 Physicochemical characteristics of cold-pressed oils

#### 3.3.1 Free fatty acids (FFA) and acid value

Across the reviewed studies, cold-pressed oils exhibited consistently low free fatty acid (FFA) content, generally ranging from 0.2 to 2% depending on botanical origin and extraction conditions (26, 27). Oils such as olive, avocado, almond, rapeseed, and groundnut typically showed low FFA values (<1%), indicating minimal hydrolytic degradation and high-quality pressing practices (28, 29, 215). Even highly sensitive oils, such as flaxseed and hempseed, maintained FFA levels within acceptable limits (0.8–2.0%) (30, 31), suggesting that cold pressing effectively preserves hydrolytic stability.

Conversely, some oils known for high enzymatic activity such as cumin seed oil (32), coriander oil (33), and cinnamon oil (34) exhibited higher FFA values, likely due to the presence of volatile constituents, seed microflora, or pre-pressing enzymatic activity. Overall, the low FFA content observed in most cold-pressed oils demonstrates the efficiency of cold pressing in minimizing lipase activation and maintaining oil freshness.

#### 3.3.2 Peroxide value (PV)

The peroxide value (PV), a marker of primary lipid oxidation, varied substantially across oils but remained within acceptable quality thresholds. Stable oils such as coconut, olive, palm, mustard, and groundnut typically exhibited PVs ranging between 1 and 8 meq O₂/kg (26, 35, 36). In contrast, PUFA-rich oils such as flaxseed, chia seed, hempseed, walnut, grape seed, and pomegranate seed presented higher PVs (6–15 meq O₂/kg), reflecting their greater susceptibility to oxidative initiation (37–39).

Despite these variations, nearly all values remained below internationally accepted limits (10–20 meq O₂/kg), indicating that cold pressing generally avoids excessive thermal or oxidative stress. Differences in PV were strongly associated with fatty acid composition, antioxidant content, and storage conditions reported in the included studies (40).

#### 3.3.3 Iodine value (IV)

The iodine value (IV), reflecting the degree of unsaturation, showed a broad range across oils. PUFA-rich oils including flaxseed (170–190), chia (180–220), hempseed (160–170), walnut (145–160), and pomegranate seed oils (130–150) recorded the highest IVs, consistent with high proportions of linoleic, alpha-linolenic, and conjugated linolenic acids (3, 41, 42). MUFA-dominated oils such as olive (80–88), avocado (85–95), and argan oil (95–105) showed moderate IV values (4, 43, 44). Oils with high saturated fatty acid (SFA) content, such as coconut and palm oil, had very low IVs (6–55), reflecting their oxidative resistance (6, 45).

These findings align with the biochemical expectations of lipid unsaturation: oils with more double bonds exhibit higher iodine consumption and greater oxidative susceptibility.

#### 3.3.4 Saponification value (SV)

Saponification values varied based on fatty acid chain length. Short- and medium-chain triglyceride oils such as coconut and palm kernel exhibited higher SV (250–265 mg KOH/g), consistent with the abundance of C8–C12 fatty acids (6). In contrast, oils richer in long-chain fatty acids such as chia, grape seed, rapeseed, and avocado demonstrated lower SV values (170–200 mg KOH/g) (39, 43). SV data indicate that cold-pressed oils maintain their intrinsic fatty acid structural profiles, allowing oil-specific functional characteristics to be preserved. The physicochemical parameters of some selected cold-pressed oils are presented in Table 1.

**Table 1:** Key physicochemical parameters of selected cold-pressed oils.

Oil type	Source category	Free fatty acids (FFA%)	Peroxide value (PV, meq O₂/kg)	Iodine value (IV, g I₂/100g)	Saponification value (SV, mg KOH/g)	Unsaponifiable matter (% w/w)	Specific gravity (25°C)	References
Argan oil	Nut	0.2–0.8	02–08	95–105	188–195	0.6–1.0	0.910–0.915	(44)
Avocado oil	Fruit	0.3–1.0	03–08	85–95	185–195	0.8–1.2	0.909–0.915	(43)
Almond oil	Nut	0.02–0.06	<03	90–105	180–195	0.3–3	0.910–0.920	(28)
Amaranthus seed oil	Seed	<2.0	2.17–3.89	78–140	160.82	2.57	0.92	(84)
Borage oil	Seed	0.91	8.12	120–160	184–195	0.39–0.78	0.910–0.940	(85, 213, 216)
Coconut oil	Fruit	0.1–0.8	01–05	6–11	250–265	0.2–0.5	0.915–0.920	(26)
Camelina oil	Seed	–	0.8–4.6	110–165	189.07–189.66	0.68–0.87	0.920–0.950	(86, 223)
Chia seed oil	Seed	1.03–1.7	1.0–3.8	180–220	197	1.12	0.93	(39)
Cinnamon oil	Bark	0.5–10	74.99	6.11–506.15	214.75–228.19	2	1.01–1.06	(34)
Coriander oil	Seed	1.8	0.92	44–82	185–215	1	0.863–0.875	(33)
Cumin seed oil	Seed	7.49	4.2–15.8	98–132	185–205	1.2–4.78	0.890–0.940	(32)
Date palm seed oil	Seed	0.5–0.9	01–08	48.84–60.59	198–228	0.62–8.92	0.88–0.94	(87)
Evening primrose oil	Seed	<4	10	125–165	187.35	01–03	0.91–0.93	(88)
Flaxseed oil	Seed	0.8–2.0	03–08	170–190	188–195	0.9–1.2	0.926–0.930	(30)
Grape seed oil	Seed	0.35–2.65	<3	94–157	180–200	0.8–2	0.91–0.93	(89)
Groundnut oil	Nut	0.3–1.0	<3	90–105	188–196	0.6–1.0	0.914–0.918	(27)
Hempseed oil	Seed	1.2–2.0	06–12	160–170	190–195	1.1–1.4	0.918–0.922	(31)
Kalonji (Black seed) oil	Seed	0.8–1.8	04–08	120–140	188–194	1.2–1.6	0.918–0.924	(90)
Milk thistle oil	Seed	0.4–1.5	1.0–5.0	95–110	185–195	0.8–2.5	0.920–0.930	(91, 210)
Mustard oil	Seed	0.5–1.0	02–06	95–110	168–176	0.7–1.0	0.912–0.917	(36, 224)
Niger	Seed	0.5–1.2	<10.0	115–135	185–195	0.8–1.5	0.925–0.930	(92)
Olive oil	Fruit	0.5–1.5	05–15	80–88	190–196	1.0–1.5	0.910–0.916	(29, 211, 212)
Orange oil	Fruit	2.3–7.83	13.4–16	75.68–120.10	185–199	0.53–5.31	0.842–0.846	(18, 226, 227)
Palm oil	Fruit	0.2–1.0	01–05	50–55	190–205	0.3–0.6	0.895–0.899	(35)
Pomegranate seed oil	Seed	1.0–1.6	03–08	130–150	185–195	1.1–1.5	0.917–0.921	(37)
Pumpkin seed oil	seed	1.0–1.8	04–10	110–125	186–193	1.0–1.5	0.912–0.918	(40)
Rapeseed (Canola) oil	Seed	0.2–1.0	02–06	110–125	170–178	0.5–0.9	0.912–0.917	(93)
Raspberry seed oil	Seed	0.8–3.0	<10.0	150–180	175–190	1.0–3.0	0.920–0.935	(38, 217)
Rosehip oil	Fruit	0.8–3.0	<10.0	150–170	180–195	0.5–1.5	0.920–0.940	(94, 225)
Rosemary oil	Leaf	<3.0	<5	82–100	—	<1	0.897	(95, 214)
Rice bran oil	Grain	1.0–4.0	<10.0	90–115	180–195	2.0–5.0	0.910–0.925	(18)
Sesame oil	Seed	0.5–1.0	03–10	104–112	186–194	0.8–1.0	0.916–0.920	(96, 218, 220, 222)
Sunflower oil	Seed	0.4–1.2	04–12	120–145	188–194	0.8–1.2	0.918–0.922	(97)
Walnut oil	Nut	0.6–1.5	04–10	145–160	188–193	1.0–1.3	0.921–0.925	(57)

#### 3.3.5 Unsaponifiable matter and specific gravity

Unsaponifiable matter-consisting of sterols, tocopherols, squalene, carotenoids, and other minor compounds-was generally higher in oils such as sesame (0.8–1.0%), argan (0.6–1.0%), pumpkin seed (1.0–1.5%), and olive (1.0–1.5%) (5, 29, 46, 47). These components are critical for biological activity and oxidative protection.

Specific gravity values ranged between 0.89 and 0.94 for most oils. Oils with high resinous or aromatic components (e.g., cinnamon, coriander) exhibited slightly higher densities (1.01–1.06), whereas citrus seed oils showed lower values (~0.842) (18, 34).

### 3.4 Stability and shelf life

The extraction method, quality of the raw material, storage conditions, and the presence of antioxidants all contribute to determining the stability and shelf life of cold-pressed oils. Not applying heat treatment means more bioactive compounds are captured. However, cold-pressed oils are more susceptible to oxidation and are more vulnerable to the presence of PUFAs. Exposure to elevated temperatures increases the susceptibility of PUFA-rich oils to oxidation; therefore, heat treatments such as roasting may accelerate lipid degradation and adversely affect oil quality.

Rancidity is triggered by free radicals and peroxides, resulting in the loss of flavor, aroma, and nutritional value in the oil (48). Close-packed polyunsaturated oils (PUFAs), such as those found in flaxseed and sunflower oil, have shorter shelf lives. Low PUFAs oil, when stored in close-packed conditions, becomes rancid and of lower quality, leading to increased rancidity and degradation. In contrast, Oils rich in monounsaturated fats, such as olive and avocado oil, will have a decrease in oxidation and an increase in shelf life (218, 222, 227).

Some strategies have been proven effective in extending the shelf life of cold-pressed oils. Using dark glass containers to store cold-pressed oils can protect them from light, a factor that contributes to the oxidation of oils. Additionally, the longitudinal preservation of the oils is facilitated by storing them in dry, cool areas away from heat sources. Some studies have suggested that incorporating the antioxidant vitamin E could further enhance the shelf life of cold-pressed oils, as it would be beneficial in the oxidation process of the oils (49, 50, 218, 222, 227). However, given that cold-pressed oils are more susceptible to oxidation, the shelf life of cold-pressed oils will always be shorter than that of refined oils. This is because oxidized oils have been processed to remove impurities and stabilize the oil.

The oxidation of cold-pressed oils over time can be quantified by the peroxide value, which determines the degree of oxidation of the oil. This demonstrates that cold-pressed olive oil oxidizes at a significantly faster rate than refined olive oil, highlighting the challenges in stabilizing cold-pressed oils.

### 3.5 Strategies to improve stability

Several strategies can greatly improve the stability of cold-pressed oils (particularly those containing polyunsaturated fatty acids). Lowering heat, light, and oxygen exposure during extraction, processing, and storage minimizes oxidative degradation. Natural antioxidants (like tocopherols, polyphenols) and plant extracts (rosemary, basil, sage) are able to block the process of lipid oxidation and thus prolong shelf life, as well as preserve nutritional value. As oilseeds are processed (through microwave heating and mild roasting) to produce Maillard reaction products (MRPs) and advanced lipid oxidation end-products (ALEs), which exhibit antioxidant activities and are associated with the stability of the oil. Moreover, packaging optimization through the use of dark-colored, airtight containers and refrigeration or nitrogen-flushed storage of oils will further reduce oxidation risks. The combination of these approaches, with careful handling of processing conditions, yields increased oxidative stability and bioactivity in cold-pressed oils, as well as enhanced potential for long-term storage and utilization in health applications (49, 51).

### 3.6 Nutritional composition

Unlike cooking oils, cold-pressed oils are rich in natural fats and vitamins, offering antioxidant benefits without the use of heat or chemical refining (211, 213, 219, 221, 223, 224). These oils typically contain a balanced profile of healthy fatty acids, including monounsaturated (MUFA) and polyunsaturated fatty acids (PUFA), which are essential for cardiovascular health and anti-inflammatory functions (211, 223, 224). Oleic acid (MUFA) from cold-pressed oils like olive and avocado oils improves lipid profiles, while essential omega-3 and omega-6 PUFAs from flaxseed and hemp oils support neurological function and reduce inflammation. These oils also preserve bioactive molecules, including tocopherols and phytosterols, which have antioxidant effects on metabolism and immunity (221). Cold-pressed oils are recognized for their low saturated fat content and high nutrient density, which support healthy diets and therapeutic applications. Table 2 summarizes the nutritional composition of some selected cold-pressed oils.

**Table 2:** Nutritional composition of selected cold-pressed oils.

Oil type	Monounsaturated fatty acids	Polyunsaturated fatty acids	Saturated fatty acids	Vitamins	Others	Reference
Almond oil	66%	<1%	8%	Tocopherols present	TCC = 6 mg BCE/kg; TPC = 1.68 mg GAE/100 g; TFC = 0.03 mg CE/g; Antioxidant = 63 mg TE/kg; P = 216 ppm; Anti-inflammatory = 60%	(98)
Amaranthus seed oil	—	—	—	Tocopherols	Squalene (≈ 65 mg/100 g), Phytosterols	(98)
	—	—	—	Tocopherols	Squalene 6–8 %, Phytosterols	(58)
	—	—	—	Tocopherols	Squalene 6–8 %	(2)
	—	—	—	—	Squalene 6–8%	(99)
Borage oil	—	α-Linolenic acid 16–27%	—	Vitamin E (1,410.2 mg/kg)	Chlorophylls (2.8 mg/kg)	(99)
Camelina oil	Eicosenoic acid 15%	Linoleic acid 30–40%	~8%	Vitamin E (713.6 mg/kg)	Sterols (510.9 mg/100 g), Carotenoids (160.1 mg/kg)	(99)
	Eicosenoic 15 %, Oleic 20 %	Linoleic 20 %, Linolenic 30 %	8–10 %	γ-Tocopherol dominant	Phytosterols, Carotenoids, Phenolics	(58)
	Oleic ≈ 20 %, Eicosenoic ≈ 15 %	Linoleic ≈ 20 %, Linolenic ≈ 30 %	8–10 %	γ-Tocopherol	Phytosterols, Carotenoids	(98)
	17.10%	66.6 % (LA 17.4 %, ALA 49.9 %)	9.90%	α n.d., γ-817.7, δ-126 mg/kg (Total 972 mg/kg)	Phenols 117.3 mg/kg; Flavonoids 16.7 mg/kg; Phytosterols 2,533 mg/kg (β-sitosterol 1,440 mg/kg); Squalene 22 mg/kg	(1)
	Eicosenoic acid 15 %	Linoleic acid 30–40 %, Linolenic acid ≈ 15 %	≈ 9 %	Tocopherols (Vitamin E)	Sterols (510.9 mg/100 g), Carotenoids (160.1 mg/kg)	(2)
	17.10%	66.6% (LA 17.4%, ALA 49.9%)	9.90%	Total 972.3 (γ 817.7 predominant)	Phenols 117.3 mg/kg; Flavonoids 16.7 mg/kg; Phytosterols 2,533 mg/kg (β-sitosterol 1,440 mg/kg); Squalene 22.2 mg/kg	(1)
	35.33 % (eicosenoic 14.43 %)	49.67 % (linoleic 19.38 %, linolenic 30.29 %)	9.72%	Total 76.98 mg/100 g (α-1.20, γ-74.27, δ-1.51)	Sterols 479 mg/100 g (β-sitosterol 265, brassicasterol 22), TPC 4.17 mg GA/100 g	(100)
Chia seed oil	Low MUFA (~7–10%) mainly oleic acid	Very high PUFA (60–70%) — α-linolenic acid (≈ 62%) and linoleic acid (~ 18%)	≈ 10–12% (SFA)	Tocopherols (Vitamin E); polyphenols present	TPC = 2.88 mg GAE/g; TFC = 0.71 mg QE/g; TCC = 2.81 mg/kg; Antioxidant activity (DPPH) = 66.33%; Induction time = 5 h; Peroxide value = 0.80 meq O₂/kg; Acid value = 2.07 mg KOH/g — rich in omega-3 ALA (ω-6/ω-3 ≈ 0.33); hypocholesterolemic/hypercholesterolemic ratio = 11.31 – 14.06 (↑ health benefit)	(101)
Cinnamon oil	Cinnamaldehyde (68–89%) unsaturated aldehyde analog of MUFA	1,8-Cineole (2.0%), α-Terpineol (0.9%)	Minor acetic acid esters (~1.8%), α-Pinene (1.5%)	Vitamin C (used as antioxidant standard in study)	Antidiabetic (↓ glucose, TG, cholesterol); anti-inflammatory via TLR-4 suppression; antimicrobial and antitumor properties	(102)
Coconut oil	8%	5%	87%	High vitamin E (tocopherols)	TCC = 3 mg BCE/kg; TPC = 5.31 mg GAE/100 g; TFC = 1.44 mg CE/g; Antioxidant = 80.75 mg TE/kg; P = 210 ppm; Anti-inflammatory = 42%	(25)
Coriander oil	Linalool (66.1%) monoterpenoid alcohol	Camphor (8.3%), Geranyl acetate (6.9%), Cymene (6.4%)	Trace long-chain terpenes (<5%)	—	Antioxidant (51% radical scavenging), antibacterial (*B. subtilis*), anthelmintic (larval cuticular damage); improves fish immunity when fed 1% oil in diet	(102)
Cumin seed oil	Cuminaldehyde (49.9%) unsaturated aromatic aldehyde	Terpenes and phenolics (~30%)	Saturated fatty chain residues (~20%)	—	Anti-inflammatory (suppresses neutrophil activation IC₅₀ = 3.8–17 μg/mL); ↓ diastolic BP in metabolic syndrome patients	(102)
	Not specified	Not specified	Not specified	—	Very high seedcake residue (94%); low oil recovery	(24)
Date palm seed oil	High MUFA (~ 40–45%) — oleic acid dominant	Low PUFA (~ 10%) — linoleic acid minor	High SFA (40–50%) — lauric (10–22%), palmitic, myristic acids	Tocopherols (Vitamin E) and β-carotene (vitamin A precursor)	TPC = 3.26 mg GAE/g; TFC = 0.89 mg QE/g; TCC = 11.24 mg/kg (β-carotene ≈ 80%); Antioxidant activity (DPPH) = 71.32%; Induction time = 34.6 h; Acid value = 1.06 mg KOH/g; Peroxide value = 1.26 meq O₂/kg — rich in phenolics and sterols; excellent oxidative stability & shelf-life enhancer	(101)
Evening primrose oil	—	Linoleic acid 70–75%, α-Linolenic acid 8–14%	—	—	Gamma-linolenic acid (GLA)	(99)
Flaxseed oil	Oleic acid ~20%	α-Linolenic acid 51.8–60.4%, Linoleic acid 15.2–17.4%	~9%	Vitamin E (Tocopherols)	Plastochromanol-8 (34.8–55.3 mg/kg), Sterols (475.4 mg/100 g), Carotenoids (147.5 mg/kg)	(99)
	Oleic 15–20 %	α-Linolenic 50–60 %, Linoleic 15–20 %	≈ 10 %	γ-Tocopherol	Lignans (Secoisolariciresinol 257 mg/100 g), Phenolics	(98)
	20.50%	68.8 % (LA 15.2 %, ALA 53.1 %)	10.70%	α-63.5, γ-540.3 mg/kg (Total 589 mg/kg)	Phenols 55.8 mg/kg; Flavonoids 15.4 mg/kg; Phytosterols 5,172 mg/kg (β-sitosterol 1,839 mg/kg); Squalene n.d.	(1)
	Oleic acid ≈ 20 %	α-Linolenic acid 51.8–60.4 %, Linoleic acid 15.2–17.4 %	≈ 10 %	Tocopherols (Vitamin E)	Plastochromanol-8 (34.8–55.3 mg/kg), Sterols (475.4 mg/100 g), Carotenoids (147.5 mg/kg)	(2)
	20.50%	68.8% (LA 15.2%, ALA 53.1%)	10.70%	Total 588.7 (γ 540.3)	Phenols 55.8 mg/kg; Flavonoids 15.4 mg/kg; Phytosterols 5,172 mg/kg (β-sitosterol 1,839 mg/kg); Squalene not detected	(1)
	Oleic acid ≈ 20%	α-Linolenic acid ≈ 50–55%, Linoleic ≈ 15%	~10%	Tocopherols (Vitamin E)	Oil yield (27.7%); lowest peroxide (2.37 meq O₂/kg); rich in omega-3 fatty acids	(24)
Grape seed oil	Oleic acid ~16%	Linoleic acid 50.1–77.8%	~10%	—	Polyphenols, Proanthocyanidins	(99)
	—	Linoleic acid 50–78 %	—	—	Sterols, Carotenoids	(2)
Groundnut oil	40%	50%	10%	Tocopherols present	TCC = 9 mg BCE/kg; TPC = 3.98 mg GAE/100 g; TFC = 0.59 mg CE/g; Antioxidant = 47 mg TE/kg; P = 311 ppm; Anti-inflammatory = 25%	(25)
Hemp seed oil	Oleic acid ≈ 12–15%	Linoleic acid ≈ 55%, α-Linolenic ≈ 20%	~10%	Tocopherols (Vitamin E)	Oil yield (20.3%); balanced omega-6/omega-3 ratio; peroxide 9.0 meq O₂/kg	(24)
High-oleic rapeseed oil (100 °C roasted)	Oleic acid 74.25%	Linoleic 11.32%, α-linolenic 5.04%	6.40%	γ-T 37.48, α-T 20.48 mg/100 g (total 57.96 mg/100 g; Vit. E = 24.23 mg α-TE/100 g)	Total sterols 610.6 mg/100 g; β-sitosterol 284.2, campesterol 224.8, brassicasterol 82.9 mg/100 g; carotenoids 11.2 mg/kg; chlorophylls 8.1 mg/kg	(22)
	Oleic acid 74.20%	Linoleic 11.32%, α-linolenic 5.04%	6.40%	γ-T 37.75, α-T 23.00 mg/100 g (total 60.75 mg/100 g; Vit. E = 26.78 mg α-TE/100 g)	Total sterols 604.1 mg/100 g; β-sitosterol 281.2, campesterol 220.3, brassicasterol 84.3 mg/100 g; carotenoids 12.6 mg/kg; chlorophylls 9.0 mg/kg	(22)
	Oleic acid 73.89%	Linoleic 11.52%, α-linolenic 5.09%	6.40%	γ-T 38.04, α-T 24.68 mg/100 g (total 62.72 mg/100 g; Vit. E = 28.48 mg α-TE/100 g)	Total sterols 626.0 mg/100 g; β-sitosterol 292.5, campesterol 227.8, brassicasterol 86.9 mg/100 g; carotenoids 14.2 mg/kg; chlorophylls 13.0 mg/kg	(22)
	Oleic acid 74.07%	Linoleic 11.41%, α-linolenic 5.08%	6.40%	γ-T 35.19 mg/100 g, α-T 15.52 mg/100 g (total 50.71 mg/100 g; Vit. E = 19.04 mg α-TE/100 g)	Total sterols 601.4 mg/100 g; β-sitosterol 281.7, campesterol 219.8, brassicasterol 82.1 mg/100 g; carotenoids 10 mg/kg; chlorophylls 6 mg/kg	(22)
	Oleic acid 74.59%	Linoleic acid 11.15%, α-linolenic 4.89%	6.4% (mainly palmitic and stearic acids)	Tocopherols: γ-T 30.17 mg/100 g, α-T 17.25 mg/100 g (total 47.42 mg/100 g; Vitamin E = 20.47 mg α-TE/100 g)	Sterols: β-sitosterol 279.6, campesterol 216.1, brassicasterol 82.7 mg/100 g; total sterols 596.5 mg/100 g; carotenoids 8.0 mg/kg; chlorophylls 3.3 mg/kg	(22)
Lemon oil	Limonene (~39–40%), Citral (neral + geranial ≈ 30%) – monoterpenes with MUFA-like unsaturation	Minor oxygenated terpenoids (β-pinene ≈ 25%) providing antioxidant synergy	Trace long-chain terpenoids (~5%)	Vitamin C-analog compounds (ascorbic acid used as control in bioassays)	IC₅₀ (DPPH) ≈ 10.2 mg/mL; strong antibacterial activity vs *C. albicans*, *L. monocytogenes*; enhances gut microbiota diversity via limonin metabolite	(102)
Linseed (Flaxseed) oil	Oleic 15–20 %	α-Linolenic 50–60 %, Linoleic 15–20 %	10%	γ-Tocopherol	Phospholipids (lecithin, cephalin), Phytosterols, Phenolics, Pigments	(58)
	20.05%	68.42 % (α-linolenic 52.12 %, linoleic 16.30 %)	11.63%	Total 44.04 mg/100 g (α-1.78, γ-42.26)	Sterols 335 mg/100 g (β-sitosterol 166), TPC 2.93 mg GA/100 g	(100)
Milk thistle oil	22.70%	58.4 % (LA 57.4 %, ALA 1.0 %)	17.40%	α-204.1, γ-55.5, δ-14.6 mg/kg (Total 262 mg/kg)	Phenols 78.8 mg/kg; Flavonoids 4.5 mg/kg; Phytosterols 3,421 mg/kg (β-sitosterol 1,480 mg/kg); Squalene 65 mg/kg	(1)
	22.70%	58.4% (LA 57.4%, ALA 1.0%)	17.40%	Total 262.0 (α 204.1 main form)	Phenols 78.8 mg/kg; Flavonoids 4.53 mg/kg; Phytosterols 3,421 mg/kg (β-sitosterol 1,479 mg/kg); Squalene 65.4 mg/kg	(1)
	Oleic acid ≈ 22%	Linoleic acid ≈ 58%	~17%	Tocopherols (Vitamin E)	Oil yield (14.5%); moderate acid value (5.27 mg KOH/g); phytosterols present	(24)
	25.69%	56.76 % (linoleic 56.46 %)	19.53%	Total 46.09 mg/100 g (α-38.91, β-2.84)	Sterols 509 mg/100 g (β-sitosterol 192), TPC 5.42 mg GA/100 g	(100)
Mustard oil	59%	32%	8%	Moderate vitamin E	TCC = 19 mg BCE/kg; TPC = 10.26 mg GAE/100 g; TFC = 0.62 mg CE/g; Antioxidant = 64 mg TE/kg; P = 176 ppm; Anti-inflammatory = 20%	(25)
Niger oil	14%	68%	18%	Moderate vitamin E	TCC = 13 mg BCE/kg; TPC = 7.21 mg GAE/100 g; TFC = 0.72 mg CE/g; Antioxidant = 59 mg TE/kg; P = 243 ppm; Anti-inflammatory = 13%	(25)
Olive oil	Oleic acid (n-9) 55–83%	—	~14%	Vitamin E (Tocopherols)	Polyphenols, Carotenoids	(99)
	Oleic acid (n-9) 55–83 %	Linoleic 3.5–21 %, Linolenic < 1 %	Palmitic 7.5–20 %, Stearic 0.5–5 %	α-Tocopherol (dominant form)	Phytosterols (β-sitosterol, campesterol, stigmasterol), Squalene (0.2–0.7 %), Phenolics, Carotenoids, Chlorophylls	(58)
	Oleic 55–83 %	Linoleic 3.5–21 %	7.5–20 %	α-Tocopherol	Phytosterols (≈ 290 mg/100 g), Squalene (200–700 mg/100 g), Polyphenols	(98)
	Oleic acid (n-9) 55–83 %	Linoleic acid (n-6) 3.5–21 %	Palmitic 7.5–20 %, Stearic 0.5–5 %	Tocopherols (Vitamin E)	Phenolic compounds, Carotenoids, Sterols, Squalene	(2)
Orange oil	Limonene (89–98%) major monoterpene	Myrcene (3.1–3.2%), β-pinene (0.55%), citral minor PUFA-like components	α-Pinene (0.3%), octanol (0.4%)	Provitamin A carotenoid traces	Strong antioxidant and antipathogenic activity; inhibits biofilm formation (*S. aureus*, *P. aeruginosa*); modulates gut Lactobacillus abundance	(102)
Palm oil	41%	9%	50%	Rich in carotenoids & tocotrienols	TCC = 812 mg BCE/kg; TPC = 22.56 mg GAE/100 g; TFC = 1.58 mg CE/g; Antioxidant = 32.65 mg TE/kg; P = 472 ppm; Anti-inflammatory = 66%	(25)
Parsley oil	Myristicin (36%), Apiole (21%) — allylbenzene MUFA analogs	α-Pinene (15%), β-Pinene (10%) — polyunsaturated terpenes	Minor alkyltetramethoxybenzenes (6.5%)	—	Highest antioxidant capacity (DPPH 64%, FRAP 0.93 mmol TE/L) but pro-oxidative in TBARS test	(102)
Pumpkin seed oil	30.80%	50.7% (LA 49.9%, ALA 0.6%)	18.20%	Total 290.8 (γ 201.4)	Phenols 106.6 mg/kg; Flavonoids 64.2 mg/kg; Phytosterols 5,460 mg/kg (mainly Δ7-sterols); Squalene 1,324.3 mg/kg	(1)
	Oleic acid ~33%	Linoleic acid ~47%	~20%	—	Squalene 0.9%, Phytosterols	(99)
	Oleic 35 %	Linoleic 50 %	15%	α- and γ-Tocopherols	Phytosterols, Squalene (0.03 %), Carotenoids (β-carotene, lutein), Chlorophyll	(58)
	Oleic ≈ 35 %	Linoleic ≈ 50 %	≈ 15 %	α-, γ-Tocopherols	Carotenoids (Lutein 1.5–9.6 mg/100 g; Zeaxanthin 2.1–8.7 mg/100 g), Phenolic acids (Caffeic, Ferulic, Coumaric), Phytosterols	(98)
	30.80%	50.7 % (LA 49.9 %, ALA 0.6 %)	18.20%	α-51.2, γ-201.4, δ-21.9 mg/kg (Total 291 mg/kg)	Phenols 106.6 mg/kg; Flavonoids 64.2 mg/kg; Phytosterols 5,460 mg/kg (mainly Δ^7^-sterols); Squalene 1,324 mg/kg	(1)
	—	Linoleic acid ≈ 50 %	—	Tocopherols (Vitamin E)	Squalene ≈ 0.9 %, Carotenoids	(2)
	Oleic acid ~35%	Linoleic acid ~50%	~15%	Tocopherols (Vitamin E)	Moderate oil yield (13.4%); highest peroxide value (18.45 meq O₂/kg); high flavonoid content; squalene present	(24)
	30.70%	50.59 % (linoleic 50.39 %)	18.92%	Total 64.45 mg/100 g (α-7.49, γ-56.96)	Sterols 300 mg/100 g (β-sitosterol 182), TPC 8.32 mg GA/100 g	(100)
Rapeseed oil	Oleic acid ~60%	α-Linolenic acid (omega-3) 9–11%	~7%	Vitamin E (Tocopherols)	Brassicasterol (104 mg/100 g), Plastochromanol-8 (55–80 mg/100 g), Sterols	(99)
	Oleic 60–65 %	Linoleic 20 %, Linolenic 10 %	6–8 %	α- and γ-Tocopherols	Phytosterols (Brassicasterol, Sitosterol), Phenolics, Carotenoids, Chlorophylls	(58)
	Oleic 60–65 %	Linoleic ≈ 20 %, Linolenic ≈ 10 %	≈ 7 %	α-, γ-Tocopherols	Phytosterols (≈ 890 mg/100 g), Carotenoids (Lutein), Phenolics	(98)
	Oleic acid ≈ 60 %	Linoleic ≈ 20 %, Linolenic ≈ 10 %	≈ 7 %	Tocopherols (α-, γ-)	Brassicasterol (104 mg/100 g), Plastochromanol-8 (55–80 mg/100 g), Sterols	(2)
	64.83 % (oleic 63.26 %)	27.94 % (linoleic 19.82 %, linolenic 8.12 %)	7.12%	Total 70.21 mg/100 g (α-27.00, γ-42.11, δ-1.10)	Sterols 684 mg/100 g (β-sitosterol 336, brassicasterol 73), TPC 4.93 mg GA/100 g	(100)
Raspberry seed oil	—	α-Linolenic acid 29.1–32.4%	—	—	Phenolic compounds	(99)
	—	α-Linolenic acid 29–32 %	—	—	Tocopherols, Sterols	(2)
Rice bran oil	Oleic acid ~38%	Linoleic acid ~37%	~25%	Vitamin E (Tocopherols, Tocotrienols)	γ-Oryzanol 1.5–2.9%	(99)
	Oleic 38 %	Linoleic 37 %	25%	Tocopherols + Tocotrienols	γ-Oryzanol (1.5–2.9 %), Squalene (0.05 %), Phytosterols	(58)
	Oleic acid ≈ 38 %	Linoleic acid ≈ 37 %	≈ 25 %	Tocopherols (α-, γ-, δ-) and Tocotrienols	γ-Oryzanols (250–490 mg/100 g; up to 2,650 mg/100 g in cold-pressed oil), Phytosterols (≈ 1,900 mg/100 g), Squalene (≈ 600 mg/100 g)	(98)
	Oleic acid ≈ 38 %	Linoleic acid ≈ 37 %	≈ 25 %	Tocopherols and Tocotrienols	γ-Oryzanol 1.5–2.9 %, Squalene	(2)
Rosehip oil	17.30%	74.6 % (LA 43.7 %, ALA 30.9 %)	7.10%	α-123.8, γ-674.8, δ-252.9 mg/kg (Total 1,036 mg/kg)	Phenols 86.8 mg/kg; Flavonoids 11.6 mg/kg; Phytosterols 5,358 mg/kg (β-sitosterol 4,314 mg/kg); Squalene 204 mg/kg	(1)
	17.30%	74.6% (LA 43.7%, ALA 30.9%)	7.10%	Total 1,036 (α 123.8, γ 674.8, δ 252.9)	Phenols 86.8 mg/kg; Flavonoids 11.6 mg/kg; Phytosterols 5,358 mg/kg (β-sitosterol 4,314 mg/kg); Squalene 203.8 mg/kg	(1)
Rosemary oil	Monoterpenes (95.6%) — α/β-Pinene, Camphene etc.	Sesquiterpenes (4.2%)	Trace resins and diterpenes (<1%)	Vitamin E (tocopherols) analog carnosic acid and rosmarinic acid present	Antioxidant, antiviral and anticancer (carnosic acid ↓ prostate cancer cell viability; rosmarinic acid ↓ Warburg effect in gastric cells)	(102)
Safflower oil	15%	74%	11%	Moderate vitamin E	TCC = 15 mg BCE/kg; TPC = 21.27 mg GAE/100 g; TFC = 0.27 mg CE/g; Antioxidant = 72 mg TE/kg; P = 216 ppm; Anti-inflammatory = 23%	(25)
Sesame oil	41%	44%	15%	Tocopherols high	TCC = 14 mg BCE/kg; TPC = 30 mg GAE/100 g; TFC = 1.27 mg CE/g; Antioxidant = 76 mg TE/kg; P = 243 ppm; Anti-inflammatory = 31%	(25)
	Oleic acid ~42%	Linoleic acid ~43%	~15%	—	Sesamin 0.5–1.1%, Lignans	(99)
	Oleic 42 %	Linoleic 43 %	15%	α-Tocopherol	Lignans (Sesamin 0.5–1.1 %), Phenolics, Phytosterols	(58)
	Oleic acid ≈ 42 %	Linoleic acid ≈ 43 %	≈ 15 %	Tocopherols (α-, γ-) and Tocotrienols	Lignans (Sesamin 538 mg/100 g; Sesamolin 133 mg/100 g; Sesaminol 102 mg/100 g), Phytosterols (≈ 640 mg/100 g)	(98)
	Oleic acid ≈ 42 %	Linoleic acid ≈ 43 %	≈ 15 %	Tocopherols (Vitamin E)	Sesamin 0.5–1.1 %, Sterols	(2)
	Oleic acid ~41%	Linoleic acid ~43%	~15%	Tocopherols (Vitamin E)	High oil yield (30.6%); low peroxide value (4.77 meq O₂/kg); phenolic antioxidants present	(24)
Sunflower oil	29.90%	58.30%	12%	Tocopherols (vitamin E)	TCC = 15 mg BCE/kg; TPC = 3.87 mg GAE/100 g; TFC = 0.16 mg CE/g; Antioxidant = 65 mg TE/kg; P = 190 ppm; Anti-inflammatory = 9%	(25)
	Oleic 25–35 %	Linoleic 55–65 %	8–10 %	γ-Tocopherol dominant	Phytosterols, Phenolics, Carotenoids (β-carotene), Chlorophyll (< 1 ppm)	(58)
	Oleic 25–35 %	Linoleic 55–65 %	8–10 %	γ-Tocopherol	Phytosterols (≈ 383 mg/100 g), Carotenoids (β-Carotene), Phenolics	(98)
	30.84%	57.74 % (linoleic 57.64 %)	11.20%	Total 75.93 mg/100 g (α-73.37, β-2.56)	Sterols 383 mg/100 g (β-sitosterol 218), TPC 5.25 mg GA/100 g	(100)
Thyme oil	Thymol (36.7%) phenolic monoterpene	*p*-Cymene (30%), γ-Terpinene (9%), Carvacrol (3.6%)	Trace hydrocarbons (~5%)	—	Antioxidant and genoprotective (↓ DNA oxidative damage); antiproliferative vs MCF-7, H460, MOLT-4 cell lines	(102)
Walnut oil	Oleic 20–25 %	Linoleic 60 %, Linolenic 10 %	10%	γ-Tocopherol	Phenolics, Phytosterols, Carotenoids	(58)
	Oleic 20–25 %	Linoleic ≈ 60 %, Linolenic ≈ 10 %	≈ 10 %	γ-Tocopherol	Phytosterols, Phenolics, Squalene	(98)
	19.10%	70.3 % (LA 58.5 %, ALA 11.7 %)	10.20%	α-48.6, γ-335.6, δ-45.6 mg/kg (Total 423 mg/kg)	Phenols 83.6 mg/kg; Flavonoids 7.6 mg/kg; Phytosterols 1,422 mg/kg (β-sitosterol 1,092 mg/kg); Squalene 58 mg/kg	(1)
	19.10%	70.3% (LA 58.5%, ALA 11.7%)	10.20%	Total 423.1 (α 48.6, γ 335.6, δ 45.6)	Phenols 83.6 mg/kg; Flavonoids 7.6 mg/kg; Phytosterols 1,421.7 mg/kg (β-sitosterol 1,091.9); Squalene 58.1 mg/kg	(1)

#### 3.6.1 Fatty acid profile

The fatty acid composition of cold-pressed oils (CPOs) largely determines their nutritional and functional properties (52). Unlike refined oils, cold-pressed varieties maintain the natural balance of saturated (SFA), monounsaturated (MUFA), and polyunsaturated fatty acids (PUFA) due to the absence of heat and chemical refining.

##### 3.6.1.1 Monounsaturated fatty acids (MUFA)

Cold-pressed oils such as olive, avocado, rapeseed, groundnut, argan and almond oil are rich in MUFAs, primarily oleic acid (C18:1) (40–80%) (4, 43, 53). Oleic acid contributes to oxidative stability and cardioprotective effects via modulation of LDL oxidation and membrane fluidity. Avocado and olive oils, in particular, demonstrated high MUFA/PUFA ratios, improving thermal stability and conferring suitability for culinary use. MUFAs are associated with improved lipid profiles, reduction in low-density lipoprotein (LDL) cholesterol, and increased high-density lipoprotein (HDL) cholesterol. These effects collectively promote cardiovascular health and reduce oxidative stress.

##### 3.6.1.2 Polyunsaturated fatty acids (PUFA)

PUFA-rich oils were dominated by omega-6 linoleic acid (LA) and omega-3 alpha-linolenic acid (ALA). Walnut, pumpkin seed, grape seed, sunflower, hemp seed, and chia oils contained 50–70% PUFA (14, 38, 39). Flaxseed was exceptional, with ALA levels exceeding 50% (54). Pomegranate seed oil uniquely contained punicic acid, a conjugated linolenic acid associated with potent anti-inflammatory and anti-obesity effects (41). These fatty acids are essential as the human body cannot synthesize them. PUFAs contribute to anti-inflammatory pathways, membrane fluidity, and neurological health (55).

The ratio of omega-6 to omega-3 fatty acids is a crucial determinant of health outcomes. Modern diets often exhibit a ratio exceeding 15:1, whereas an optimal ratio ranges from 2:1 to 5:1. Cold-pressed oils, such as flaxseed and chia seed oil, provide a favorable omega-3 content that can help restore this balance, thereby mitigating the risks of inflammation, cardiovascular disease, and metabolic syndrome (56).

##### 3.6.1.3 Saturated fatty acids (SFA)

SFA content varied widely across oils. Coconut oil demonstrated extremely high SFA levels (70–92%) primarily due to lauric, caprylic, and capric acids (6). Palm oil also showed high SFA proportions (48–55%), dominated by palmitic acid (45). Oils such as olive, almond, grape seed, sesame, sunflower, and pumpkin seed contained moderate SFA ranges (10–20%), usually comprising palmitic and stearic acids (5, 57). These variations reflect plant-specific lipid biosynthetic pathways.

### 3.7 Bioactive compounds: phenolics, tocopherols, sterols, and terpenes

Cold-pressed oils contain a wide range of bioactive compounds that contribute to various therapeutic properties (58). These include bioactive compounds such as tocopherols (vitamin E variants), phenolic constituents, sterols, carotenoids, and volatile organic compounds, all of which contribute to the therapeutic and health-improving benefits of the oils. Of these, bioactive polyphenols and antioxidants hold the greatest significance. These secondary metabolites possess potent antioxidant, anti-inflammatory, and antimicrobial properties, which positively impact health and contribute to the aroma, flavor, and sensory attributes present in the oil. The incorporation of these compounds is also crucial for protecting oils against oxidative degradation, extending shelf life, and maintaining quality. Furthermore, certain volatile compounds arising from lipid oxidation and plant origin also help in the unique organoleptic properties of cold-pressed oils. The polyphenols in cold-pressed olive oil, specifically hydroxytyrosol, function as a potent antioxidant, protecting against chronic diseases such as cancer and cardiovascular disease (59). Hydroxytyrosol also exhibits strong anti-inflammatory properties and may help treat inflammatory diseases, such as arthritis.

In addition to polyphenols, carotenoids are also found in cold-pressed sunflower and olive oils. These carotenoids, particularly lutein and zeaxanthin, are bioprotective and help prevent oxidative damage, while also promoting and improving vision. Cold-pressed oils also have tocopherols, specifically, vitamin E, which is a major bioactive constituent. Vitamin E diminishes the negative effects of free radicals by neutralizing them, which prevents oxidative damage to cells and tissues. Cold-pressed sunflower oil is highly regarded for its high vitamin E content and is recommended for improving skin health, particularly in terms of healing, hydration, and reducing inflammation. Sesame, pumpkin seed, and hempseed oils showed particularly high γ-tocopherol levels, enhancing oxidative stability (14, 60).

Phytosterols, or plant-derived sterols, constitute another class of bioactive compounds in cold-pressed oils. These compounds have been proven to lower cholesterol and are found in sesame oil and sunflower oil, which help reduce LDL cholesterol and improve cardiovascular health. Triterpenes such as squalene were prominent in olive, argan, and pumpkin seed oils (46, 61). Sterols such as β-sitosterol and campesterol were abundant in mustard, sesame, and rapeseed oils, providing lipid-modulating effects (62). Phytosterols also possess anti-inflammatory properties in addition to their cholesterol-lowering effects. The addition and unique combination of these bioactive compounds make cold-pressed oils health-promoting and scientifically justified for inclusion in every balanced diet. The bioactive compounds present in cold-pressed oils are detailed in Table 3.

**Table 3:** Major bioactive components and their chemical classes in cold-pressed edible oils.

Oil type	Bioactive component	Class of compound	Reference
Flax seed oil	Alpha-Linolenic Acid	Omega-3 Polyunsaturated Fatty Acid (PUFA)	(3)
	Linoleic Acid (LA)	Omega-6 Polyunsaturated Fatty Acid (PUFA)	(54)
	Oleic Acid	Monounsaturated Fatty Acid (MUFA)	(54)
	Lignans (e.g., Secoisolariciresinol Diglycoside - SDG)	Phytoestrogens (Phenolic Compounds)	(54)
	Tocopherols (Vitamin E)	Fat-Soluble Antioxidant	(13)
Olive oil Sunflower oil	Oleic Acid	Monounsaturated Fatty Acid (MUFA)	(103)
	Hydroxytyrosol (HT)	Phenolic Alcohol (Simple Phenol)	(4)
	Oleuropein (and its derivatives/aglycon)	Secoiridoid (Complex Polyphenol)	(4)
	Oleocanthal	Secoiridoid Derivative (Phenol)	(4)
	α-Tocopherol	Vitamin E (Lipophilic Antioxidant)	(4)
	Squalene	Triterpene (Hydrocarbon)	(4)
	α-Tocopherol	Vitamin E (Fat-Soluble Antioxidant)	(7)
	Linoleic Acid (High-Linoleic Type)	Omega-6 Polyunsaturated Fatty Acid (PUFA)	(7)
	Oleic Acid	(High-Oleic Type) Monounsaturated Fatty Acid (MUFA)	(104)
	Squalene	Triterpene (Hydrocarbon)	(104)
Sesame oil	Sesamin	Lignan (Phenolic Compound/Phytoestrogen)	(105)
	Sesamolin	Lignan (Phenolic Compound)	(5)
	Sesamol	Phenolic Alcohol (Derived from Sesamolin during processing)	(5)
	Tocopherols (γ- and δ-Tocopherols)	Vitamin E (Fat-Soluble Antioxidant)	(5)
	Phytosterols (β-Sitosterol)	Plant Sterols (Triterpenoids)	(5)
Coconut oil	Lauric Acid	Medium-Chain Saturated Fatty Acid (MCFA)	(6)
	Caprylic Acid (C8:0) & Capric Acid (C10:0)	Medium-Chain Saturated Fatty Acids (MCFAs)	(6)
	α- and γ-Tocopherols	Vitamin E (Fat-Soluble Antioxidant)	(106)
	Phytosterols	Plant Sterols (Triterpenoids)	(106)
Pumpkin Seed oil	Squalene	Triterpene	(61)
	Oleic Acid (Omega-9)	Monounsaturated Fatty Acid (MUFA)	(61)
	Tocopherols (mainly γ-Tocopherol)	Vitamin E (Fat-Soluble Antioxidant)	(14)
	Carotenoids	Tetraterpenoids (Pigments/Pro-Vitamin A)	(47)
Hemp seed oil Avocado oil	Alpha-Linolenic Acid (ALA)	Omega-3 Polyunsaturated Fatty Acid (PUFA)	(107)
	Gamma-Linolenic Acid (GLA)	Omega-6 Polyunsaturated Fatty Acid (PUFA)	(107)
	Tocopherols (mainly γ-Tocopherol)	Vitamin E (Fat-Soluble Antioxidant)	(60)
	Phenolic Compounds (e.g., Lignanamides, Hydroxycinnamic Acid Amides)	Polyphenols/Lignans	(60)
	Trace Cannabinoids (e.g., Cannabidiol - CBD)	Phytocannabinoids	(108)
	Lutein	Carotenoid (Tetraterpenoid Pigment)	(109)
	β-Sitosterol	Phytosterol (Plant Sterol)	(109)
	Oleic Acid (ώ-9)	Monounsaturated Fatty Acid (MUFA)	(15)
	Tocopherols (mainly α- and γ-Tocopherol)	Vitamin E (Fat-Soluble Antioxidant)	(15)
	Phenolic Compounds	Polyphenols (Antioxidants)	(110)
Mustard oil	β-Sitosterol	Phytosterol (Plant Sterol)	(111)
	Erucic Acid (ώ-9)	Monounsaturated Fatty Acid (MUFA)	(111)
	Allyl Isothiocyanate (AITC)	Isothiocyanate (Organosulfur Compound)	(111)
	Alpha-Linolenic Acid (ALA) (ώ-3)	Polyunsaturated Fatty Acid (PUFA)	(111)
	Glucosinolates	Glucosinolate (Sulfur-containing Glycoside)	(111)
Palm oil	Palmitic Acid	Saturated Fatty Acid (SFA)	(45)
	Tocotrienols (α, γ, δ)	Vitamin E (Fat-Soluble Antioxidant)	(112)
	β-Carotene (α and β carotene)	Carotenoid (Tetraterpenoid Pigment)	(113)
	Squalene	Triterpene (Hydrocarbon)	(113)
Walnut oil	Ellagitannins	Plant Sterols	(114)
	Linoleic Acid (LA) (ώ-6)	Polyunsaturated Fatty Acid (PUFA)	(114)
	α-Linolenic Acid (ALA) (ώ-3)	Polyunsaturated Fatty Acid (PUFA)	(42)
	γ-Tocopherol	Vitamin E (Fat-Soluble Antioxidant)	(115)
	Melatonin	Indoleamine Hormone	(115)
	Phytosterols (β-Sitosterol)	Plant Sterols (Triterpenoids)	(116)
Ground nut oil	Oleic Acid (ώ-9)	Monounsaturated Fatty Acid (MUFA)	(116)
	Linoleic Acid (LA) (ώ-6)	Polyunsaturated Fatty Acid (PUFA)	(117)
	Palmitic Acid (C16:0)	Saturated Fatty Acid (SFA)	(117)
	Phytosterols (mainly β-Sitosterol)	Plant Sterols (Triterpenoids)	(118)
	Tocopherols (Vitamin E)	Fat-Soluble Antioxidant	(118)
	Resveratrol (Trace)	Stilbenoid (Polyphenol)	(118)
Rapeseed (Canola) oil	Oleic Acid (ώ-9)	Monounsaturated Fatty Acid (MUFA)	(119)
	Linoleic Acid (LA) (ώ-6)	Polyunsaturated Fatty Acid (PUFA)	(119)
	Alpha-Linolenic Acid (ALA) (ώ-3)	Polyunsaturated Fatty Acid (PUFA)	(119)
	Phytosterols (mainly β-Sitosterol)	Plant Sterols (Triterpenoids)	(62)
	Tocopherols (Vitamin E)	Fat-Soluble Antioxidant	(62)
	Phenolic Acids (Trace)	Polyphenols	(62)
Kalonji (Black seed) oil	Thymoquinone (TQ)	Monoterpene Quinone (Volatile Oil)	(120)
	Linoleic Acid (LA) (ώ-6)	Polyunsaturated Fatty Acid (PUFA)	(120)
	Thymohydroquinone (THQ)	Monoterpene Quinone (Volatile Oil)	(120)
	Thymol	Phenolic Monoterpene (Volatile Oil)	(121)
	Nigellone	Polymerized Form of TQ (Dimer)	(121)
	Phytosterols (β-Sitosterol)	Plant Sterols (Triterpenoids)	(121)
Pomegranate seed oil	Punicic Acid (PuA)	Conjugated Linolenic Acid (CLnA)/ώ-5 PUFA	(41)
	Linoleic Acid (LA) (ώ-6)	Polyunsaturated Fatty Acid (PUFA)	(41)
	Oleic Acid (ώ-9)	Monounsaturated Fatty Acid (MUFA)	(41)
	Tocopherols (α-, γ, δ)	Vitamin E (Fat-Soluble Antioxidant)	(122)
	Phytosterols	Plant Sterols (Triterpenoids)	(122)
	Polyphenols (Trace)	Phenolic Compounds	(122)
Argan oil	Squalene	Triterpene (Hydrocarbon)	(46)
	Schottenol & Spinasterol	Phytosterols (Plant Sterols)	(46)
	Phenolic Compounds (e.g., Ferulic Acid)	Polyphenols	(123)
	γ-Tocopherol	Vitamin E (Fat-Soluble Antioxidant)	(123)
	Linoleic Acid (LA) (ώ-6)	Polyunsaturated Fatty Acid (PUFA)	(123)
Almond oil	α-Tocopherol	Vitamin E	(53)
	β-Sitosterol	Phytosterol	(53)
	Phenolic Compounds	Polyphenol	(53)
Amaranthus seed oil	Squalene	Triterpene Hydrocarbon	(124)
	β-Tocopherol	Vitamin E	(124)
	Phytosterols	Phytosterol	(124)
Borage oil	Gamma-Linolenic Acid (GLA)	Polyunsaturated Fatty Acid (ω-6 PUFA)	(125)
	Rosmarinic Acid	Phenolic Acid	(125)
Camelina oil	Alpha-Linolenic Acid (ALA)	Polyunsaturated Fatty Acid (ω-6 PUFA)	(126)
	Tocopherols	Vitamin E	(126)
	Lutein	Carotenoid	(126)
Chia seed oil	Alpha-Linolenic Acid (ALA), Caffeic Acid, Quercetin	Polyunsaturated Fatty Acid (ω-6 PUFA)	(127)
		Phenolic Acid	(127)
		Flavonoid	(127)
Cinnamon oil	Cinnamaldehyde, Eugenol	Aldehyde	(128)
		Phenol	(128)
Palm oil	Tocotrienols	Vitamin E	(129)
	β-Carotene	Carotenoid (Pro-Vitamin A)	(129)
	Phytosterols	Phytosterol	(129)

### 3.8 Comparative nutritional advantage

Cold-pressed oils have a distinct nutritional advantage over refined oils due to the preservation of unsaponifiable matter, a fraction rich in tocopherols, sterols, and phenolic compounds. The absence of refining, bleaching, and deodorization processes prevents the thermal degradation of these nutrients, thereby maintaining the oil’s aromatic and sensory profile.

Processing has a profound effect on sensory and nutritional quality. While refining extends shelf life, it also strips oils of antioxidants and natural pigments. Conversely, cold pressing retains both nutritional integrity and functional properties, making CPOs superior in terms of health benefits, despite their shorter storage stability.

### 3.9 Pharmacological activities

Cold-pressed oils exhibit significant pharmacological activities due to their rich content of bioactive compounds, including tocopherols, sterols, polyphenols, and essential fatty acids. Such oils exhibit powerful antioxidant, anti-inflammatory, and antimicrobial properties, thereby enhancing their ability to protect against oxidative stress and chronic diseases. Moreover, cold-pressed oils enhance heart health benefits by improving cholesterol profiles, providing immune support, and aiding in wound healing processes. In addition, due to their absence of harmful chemicals and the nullity of trans fats, they have excellent therapeutic potential, thus becoming attractive for nutritional and medicinal purposes.

#### 3.9.1 Antioxidant activity

Cold-pressed oils exhibit significant antioxidant activity due to their rich constituents, including bioactive compounds such as tocopherols, polyphenols, phytosterols, and carotenoids, which are generally preserved through the low-temperature mechanical extraction process. Consequently, they have been chosen for use in bioactive compounds and energy-intensifying oils. The antioxidants are responsible for enabling the oils to scavenge free radicals; thus protecting the oils from oxidation and extending their shelf life. The lipophilic fractions of cold-pressed oils exhibit a markedly increased radical scavenging activity, as demonstrated by investigations such as the DPPH (2,2-diphenyl-1-picrylhydrazyl) assay. For instance, sesame oil lipid extracts have demonstrated remarkable antioxidant activity compared to other seed oils (5). The interaction of multiple antioxidants can also contribute to the oxidative stability and health benefits of these oils, relative to refined oils, in most cases, as these oils lose many of their antioxidant activities during processing. Additionally, the antioxidant activity of cold-pressed oils is positively correlated with their phenolic and tocopherol content, which is dependent on the oil source. This antioxidant activity not only conserves the oils but may also provide some protective properties against diseases associated with oxidative stress when consumed (1, 63). Earlier research reports have shown that sunflower oil (64), Sesame oil (65), Pumpkin seed oil (8, 66), and Avocado oil (67) possess antioxidant activities. Other research has also confirmed the antioxidant activities of various cold-pressed oils, as listed in Table 4. However, these findings are primarily based on preclinical studies and require further clinical validation.

**Table 4:** Summary of pharmacological effects of selected cold pressed oils.

Oil type	Pharmacological activity	Models used in studies	References
Flaxseed	Cardioprotective	LDL receptor-deficient mice (*In vivo* model of atherosclerosis)	(73)
	Hypotensive	Human clinical trials (patients with peripheral arterial disease)	(11)
	Antioxidative	*In vitro* tests (DPPH, FRAP assays)	(10)
	Improved Reproductive Health	Human (Women undergoing IVF cycles with decreased ovarian reserve)	(130)
Oilve oil	Anti-inflammatory	*In vivo* (obese rats, mice)	(69)
	Antidiabetic	*In vivo* model (Mice with alloxan-induced diabetes)	(131)
	Anticancer	*In vitro* (Human colon adenocarcinoma cells)	(9)
Sunflower oil	Antioxidant	*In vitro* assays (DPPH radical inhibition, Oxidative stability tests)	(64)
	Skin Barrier Function	*Ex vivo* model using excised porcine skin	(132)
	Cardiovascular Health	*In vitro* assays (fatty acid profile and micronutrient content)	(100)
Sesame oil	Antioxidant	*In vitro* assays (DPPH radical inhibition, Measurement of oxidative stress markers)	(65)
	Hypocholesterolemic	*In vivo* model using Rats	(133)
	Anti-atherosclerotic	*In vivo*Atherosclerotic mouse models	(134)
Coconut oil	Anti-inflammatory	*In vitro*assayusing LPS-stimulated RAW 264.7 macrophage cells	(70)
	Antimicrobial/Antifungal	*In vitro*assay against various microbes *(Staphylococcus aureus, Bacillus cereus, Candida albicans, Propionibacterium acnes).*	(228)
	Wound Healing	*In vivo*assay using Dermal wound healing models in rats	(135)
Pumpkin seed oil	Antioxidant	*In vitro* assays (DPPH scavenging assay, ABTS assay, FRAP assay)	(8)
	Cardioprotective	Pilot studies in postmenopausal women [monitoring lipid profiles: (HDL-C and LDL-C)]	(74)
	Anti-inflammatory	*In vivo* assay using IBD rat model	(66)
Hempseed oil	Dermatological	*In vivo* studies (evaluating skin hydration, sebum, and TEWL after topical application)	(136)
	Anti-inflammatory	Double-blind prospective study in patients with Knee Osteoarthritis (KOA) using HSO combined with terpenes.	(137)
	Antioxidant Activity	*In vitro* DPPH assay and *In vivoDrosophila melanogaster* larvae model	(138)
Avocado oil	Anti-inflammatory	*In vivo*assay using Carrageenan-induced paw edema in mice	(71)
	Antioxidant	*In vitro*assays using DPPH and ABTS radical scavenging, FRAP, ORAC	(67)
	Antimicrobial/Antifungal	*In vitro* Models: Testing against bacteria (*Aspergillus niger*) and fungi (*Candida albicans*)	(139)
Mustard oil	Anti-inflammatory	*In vivo*: Human Clinical Studies (massage vs. olive oil on pain perception in arthritis)	(72)
	Antimicrobial	*In vitro* Models against *E. coli*, *S. aureus*, *C. albicans*	(140)
	Anticancer	*In vitro* using various human cancer cell lines	(141)
Palm oil	Oxidative stress reduction	*In vitro* model using mice subjected to oxidative stress	(142)
	Antimicrobial	*In vitro*assay againstPropionibacterium acnes, Staphylococcus epidermidis, Escherichia coli and Pseudomonas aeruginosa	(112)
Walnut oil	Antihypertensive	Human Clinical Trials: Intervention studies in normotensive or mildly hypertensive subjects	(12)
	Anti-inflammatory	*In vivo* and *In vitro* models using rats and mice	(143)
Groundnut oil	Antioxidant	*In vitro* antioxidant assays	(144)
	Lipid metabolism modulation	*In vivo* (Sprague–Dawley rats fed high-fat diet)	(145)
	Antimicrobial and anti-inflammatory activities	*In vitro* cell and enzyme assays	(146)
	Neuroprotective/Anti-Alzheimer’s activity	*In vitro* enzyme inhibition and *in silico* molecular docking	(79)
Rapeseed oil	Cardioprotective (Improving blood lipid profiles, anti-atherosclerotic)	Clinical Trials (Human intervention studies, e.g., on obese men, replacement of saturated fats), *In vivo* (Animal models)	(147)
	Anti-diabetic/Anti-obesity (Reducing insulin resistance, managing weight/BMI)	Clinical Trials (Human weight-maintenance programs), *In vivo* (Animal models)	(148)
	Antioxidant	*In vitro* (e.g., radical scavenging assays), Components (Vitamin E, Phenolic acids) studied in Cell lines	(148)
	Anti-inflammatory	Clinical Trials (e.g., assessing inflammatory markers in adipose tissue), *In vitro*	(149)
	Dermatological/Cosmetic (Anti-aging, moisturizing, UV protection, anti-wrinkle)	*In vitro* (e.g., inhibiting myeloperoxidase activity, cellular ROS), Topical application (Implied Clinical use in products)	(148)
Kalonji seed oil	Anti-inflammatory & Analgesic	*In vivo* (Animal models: acetic acid-induced writhing, paw edema, colitis, encephalomyelitis, arthritis), Clinical Trials (e.g., osteoarthritis patients)	(150, 151)
	Antioxidant	*In vitro* (TLC screening, free-radical scavenging, ROS reduction in cell lines like SH-SY5Y and BV-2 microglia), *In vivo* (Rats, rabbits: upregulating SOD, CAT, GSH; reducing MDA)	(150, 152)
	Anti-diabetic/Anti-hyperglycemic	Clinical Trials (Human subjects: improving insulin and blood sugar markers), *In vivo* (Animal models: boosting β-cell production)	(150)
	Anti-cancer/Antiproliferative	Cell lines (e.g., blood, breast, colon, pancreatic, liver, lung, prostate, cervix cancer cells; MC38 growth rate), *In vivo* (Animal models of lung, kidney, skin, colon, breast cancer)	(150)
	Anti-microbial & Antiviral	*In vitro* (Against Gram-positive/negative bacteria, viruses, fungi, parasites)	(150)
	Cardioprotective/Anti-hyperlipidemic	Clinical Trials (Human subjects: reducing total cholesterol, LDL, blood pressure), *In vivo*	(150)
	Gastroprotective/Anti-ulcer	*In vivo* (Rats: against gastric mucosal injury induced by ischemia/reperfusion)	(150)
Pomegranate seed oil	Antioxidant	*In vitro* (e.g., DPPH, ABTS free radical scavenging, FRAP), *In vivo* (animal models), Cell lines (e.g., HepG2)	(122)
	Anti-inflammatory	*In vivo* (animal models like high-fat diet rats), *In vitro* (e.g., breast cancer cell lines, modulation of pro-inflammatory cytokines)	(122, 153)
	Anti-cancer/Antiproliferative	Cell lines (e.g., HeLa, MCF-7, HT-29, normal fetal lung fibroblasts MRC-5), *In vivo* (animal models)	(122)
	Anti-diabetic (Reducing insulin resistance, anti-hyperglycemic)	*In vivo* (animal models, e.g., high-fat diet rats), Clinical trials (e.g., Type 2 Diabetes Mellitus patients)	(154)
	Skin Photoaging/Cosmetic (Anti-wrinkling, increasing skin elasticity/density, healing, anti-acne)	Topical application (e.g., human volunteers), *In vitro* (e.g., keratinocytes division, angiogenesis restriction)	(122)
Argan oil	Cardiovascular/Cardioprotective (Lipid metabolism, anti-atherogenic, blood pressure regulation)	Clinical trials (Human intervention studies), *In vivo* (animal models)	(155, 156)
	Anti-inflammatory	*In vivo* (animal models), *In vitro* (cell lines, e.g., limiting immune cell infiltration, decreasing pro-inflammatory biomarkers)	(156, 157)
	Antioxidant	*In vitro* (free-radical scavenging), *In vivo* (animal models, e.g., preventing DNA damage, enhancing oxidative status in platelets)	(155)
	Dermatological/Cosmetic (Anti-aging, skin elasticity improvement, hydration, anti-melanogenesis)	Clinical trials (Topical application on human volunteers), *In vitro* (cell lines, e.g., melanogenesis-related enzymes, Western blot, Real-time PCR)	(156)
	Anti-diabetic	*In vivo* (animal models)	(157)
Almond Oil	Cardiometabolic Protection (Hypolipidemic: HDL, LDL)	Clinical Trials/Human Intervention Studies (Dietary consumption in healthy men and women), *In vivo* (Animal models, e.g., rats with altered lipid profiles)	(158)
	Antioxidant	*In vitro* (DPPH, ABTS radical scavenging assays, measuring IC_50_) Cell Lines (C. elegans model, Keratinocytes), *In vivo* (Rats, *Saccharomyces cerevisiae* model under H_2_0_2_ and CCl_4_ stress)	(141)
	Anti-inflammatory	*In vitro* (BSA protein denaturation assay), *In vivo* (Xylene-induced ear edema model in rats/mice, Carrageenan-induced paw edema model in rats/mice)	(141)
	Anti-cancer/Anti-angiogenic (Inhibiting tumor blood vessel growth)	*In vitro* (Colon cancer cells), *In vivo* (Rat aorta ring assay, Chorioallantoic Membrane (CAM) assay in fertilized chicken eggs)	(158)
	Hepatoprotective (Liver protection)	*In vivo* (Rat model exposed to hepatotoxins like TAA), measuring improvement in altered lipid profiles.	(158)
	Neuroprotective/Antidepressant	*In vivo* (Rat models using Forced Swim Test and Passive Avoidance Test)	(159)
	Dermatological/Wound Healing (Emollient, moisturizer, anti-scarring, pressure injury prevention)	Clinical Trials (Topical application in patients, e.g., single-blind randomized trials for pressure injury prevention), *In vivo* (Skin fixation studies on rabbit skin)	(159)
Amaranthus seed oil	Cardioprotective/Hypolipidemic	Clinical Trials (Human subjects with Coronary Heart Disease and/or Hypertension, measuring cholesterol, LDL, and blood pressure). *In vivo* (Animal models, e.g., chickens, rats, measuring blood cholesterol).	(160)
	Dermatological/Anti-aging (Emollient, moisturizing, wound healing, UVA protection)	*In vitro* (Human Skin Fibroblast cell lines, measuring collagen biosynthesis, NF-_Κ_B expression, and COX-2 expression after UVA radiation). Topical/Cosmetic Applications (Formulation studies).	(160)
	Antioxidant	*In vitro* (DPPH/ABTS free radical scavenging assays). Cell Lines (Human skin fibroblasts, mitigating UVA-induced effects). *In vivo* (Implied in CCl_4_-induced toxicity models).	(161)
	Anti-inflammatory	*In vitro* (Human skin fibroblasts, suppressing NF-_Κ_B and COX2 expression induced by UVA). *In vivo* (Peptides derived from seeds in IgE-mediated food allergy mouse model).	(161)
	Hepatoprotective (Liver protection)	*In vivo* (Rat models, modulating cell membranes of hepatocytes and regulating lipid profile). *In vitro* (HepG2 cells against CCl_4_-induced toxicity for whole extract).	(14)
	Antimicrobial/Antifungal	*In vitro* (Against *Candida albicans* strains, testing synergistic activity with antifungal drugs like terbinafine).	(14)
Borage Oil	Anti-inflammatory (Modulating eicosanoid balance, pro-inflammatory cytokines)	Clinical Trials (Human patients with Rheumatoid Arthritis (RA), assessing joint tenderness/swelling). *In vivo* (Animal models of colitis, paw edema).	(162)
	Dermatological/Anti-eczema (Improving skin barrier function, reducing inflammation)	Clinical Trials (Human patients with Atopic Dermatitis (AD)/Eczema, assessing and scores). *In vitro* (Cell culture systems, e.g., human keratinocytes).	(229)
	Immunomodulatory (Affecting -cell function, hypersensitivity reactions)	*In vivo* (Animal models, e.g., -cell proliferation assays, transplant models). Clinical Trials (E.g., patients with systemic lupus erythematosus (SLE)).	(163, 229)
	Cardiovascular/Antithrombotic (Reducing platelet aggregation, blood pressure)	*In vivo* (Animal models, e.g., rats, assessing effects on platelet activity and blood pressure). *In vitro* (Platelet aggregation assays).	(163, 229)
	Anti-cancer/Chemopreventive (Inhibiting tumor growth and angiogenesis)	*In vitro* (Various cancer cell lines, (breast), (colon), inducing apoptosis). *In vivo* (Animal models of cancer).	(229)
	Neuroprotective (Cognitive enhancement, anxiety)	*In vivo* (Animal models, e.g., rats, assessing memory performance and anxiety tests).	(229)
Camelina oil	Cardiovascular/Lipid Profile Improvement	Human (Randomized Controlled Trials (RCTs) e.g., postmenopausal women, individuals with dyslipidemia); Rats (Growing rats, diet-fed models)	(164)
	Antidiabetic/Glucose Homeostasis	Human (RCTs, e.g., Non-Alcoholic Fatty Liver Disease (NAFLD) patients); Rats (Type 2 Diabetic (T2DM) models)	(164)
	Anti-inflammatory	Human (RCTs, e.g., NAFLD patients, older adults); *In vivo* (Animal models)	(165)
	Antioxidant	*In vitro* (DPPH, ABTS radical scavenging assays); Human (RCTs, e.g., NAFLD patients)	(165)
	Hepatoprotective	Human (RCTs, e.g., NAFLD patients); Rats (T2DM models)	(164)
Chia seed Oil	Cardiometabolic/Dyslipidemia Control	Human (Randomized Controlled Trials/RCTs, e.g., in overweight/obese individuals, those with diabetes); *In vivo* (Rats with high-fat, high-sucrose diet)	(166)
	Anti-inflammatory	Human (RCTs, on inflammatory markers like C-Reactive Protein (CRP)); *In vivo* (Rats - Adjuvant-induced arthritis model, Chronic Immobilization Stress model, Ulcerative Colitis model); *In vitro* (Macrophage-like cells, for nanoemulsion)	(166)
	Antioxidant	*In vitro* (DPPH, ABTS, -carotene linoleate system assays); *In vivo* (Rats with stress-induced neurodisturbance)	(167)
	Antidiabetic/Glucose Tolerance	Human (RCTs, e.g., in individuals with type 2 diabetes); *In vivo* (Rats with sucrose-rich diet, type 2 diabetic models)	(167)
	Hepatoprotective	*In vivo* (Rats with diet-induced obesity/steatosis)	(167)
	Anticancer/Cytotoxicity	*In vitro* (Human lymphoblastic leukemic cell lines, HeLa, and MCF-7 cells, LNcap, HepG2)	(168)
	Neuroprotective/Anti-Stress	*In vivo* (Rats - Chronic Immobilization Stress model)	(168)
Cinnamon oil	Antimicrobial (Antibacterial/Antifungal)	*In vitro* (Agar well diffusion, Micro-dilution, Bioautography against pathogens like *Staphylococcus aureus*, *E. coli*, *Salmonella*, *Candida albicans*); *In vivo* (Rats - MRSA infected wound model; Nematodes - *C. elegans* infected with *P. aeruginosa*)	(128)
	Antidiabetic/Hypoglycemic	*In vivo* (Mice - diabetic model, db/db mice; Rats - Streptozotocin (STZ) or Alloxan-induced diabetic models); *In vitro* (H4IIE rat hepatoma cells, for gluconeogenesis studies)	(128)
	Anti-inflammatory	*In vitro* (LPS-activated macrophages, human dermal fibroblast system, PMNCs); *In vivo* (Mice - DSS-induced colitis model; Rats - TNBS-induced colitis model)	(169)
	Antioxidant	*In vitro* (DPPH, ABTS assays, to measure radical scavenging activity); *In vivo* (Rats - models of oxidative stress/injury, e.g., gentamicin-induced nephrotoxicity)	(169)
	Cardiovascular/Lipid-Lowering	*In vivo* (Mice - diabetic models; Rats); Human (Clinical studies on lipid profile)	(169)
	Neuroprotective	*In vitro* (LPS-activated BV2 microglia); *In vivo* (Models relevant to Alzheimer's and Parkinson's disease)	(170)
	Anticancer	*In vitro* (Various human cancer cell lines)	(170)
Coriander Oil	Antimicrobial (Antibacterial/Antifungal)	*In vitro* (Micro-dilution, Disc-diffusion assay against pathogens like *Staphylococcus aureus*, *E. coli*, *Candida albicans*, *Bacillus subtilis*); *In vivo* (Mice induced by *S. aureus*)	(171)
	Antioxidant	*In vitro* (DPPH, ABTS, H_2_0_2_ radical scavenging assays, Lipid peroxidation inhibition); *In vivo* (Mice and Rats liver homogenate, stress models)	(171)
	Antidiabetic/Hypoglycemic	*In vivo* (Rats - models of diabetes, e.g., STZ-induced)	(172)
	Gastrointestinal/Digestive	Traditional/Folk Use (Carminative, spasmolytic); *In vivo* (Models for gastrointestinal disturbances)	(172)
	Analgesic/Anti-inflammatory	*In vivo* (Mice - Acetic acid-induced writhing and tail-flick methods); *In vitro* (Human dermal fibroblast system)	(173)
	Cardiovascular/Diuretic	*In vivo* (Rats - antihypertensive models)	(173)
Cumin Seed Oil	Antidiabetic/Hypoglycemic	Human (Randomized Double-Blind Placebo-Controlled Clinical Trial on Type 2 Diabetes patients); *In vivo* (Rats - Streptozotocin (STZ) or High Fructose/STZ-induced diabetic models); *In vitro* (-Amylase/-Glucosidase inhibition assays, L6 Myotubes)	(174)
	Cardiovascular/Antidyslipidemic	Human (RCTs on Type 2 Diabetes patients); *In vivo* (Rats - High-fat diet, STZ-induced models)	(174)
	Anti-inflammatory	Human (Clinical Trials on Type 2 Diabetes patients - measuring TNF-, hsCRP); *In vivo* (Rats - diabetic models)	(175)
	Antioxidant	*In vitro* (Assays, e.g., DPPH, to measure radical scavenging activity); *In vivo* (Rats - diabetic models, measuring MDA, etc.)	(175)
	Antimicrobial (Antibacterial)	*In vitro* (Against various pathogens, e.g., *S. aureus*); *In vivo* (Rats - *S. aureus* infected wound model in diabetic rats)	(175)
Date Palm seed oil	Antioxidant	*In vitro* (DPPH, ABTS radical scavenging assays to measure radical scavenging ability); *In vivo* (Human - Middle-aged women, measuring inflammation/oxidative stress markers in blood)	(176)
	Anti-inflammatory	*In vitro* (Human skin in an H_2_O_2_-induced oxidative stress model); Clinical (Human - Middle-aged women, measuring expression of IL-1β, COX-1, COX-2)	(177)
	Antidiabetic/Hypoglycemic	*In vivo* (Rats - High-fructose diet model); Traditional/Folk Use	(178)
	Antimicrobial (Antibacterial/Antiviral)	*In vitro* (Against pathogens like Methicillin-Resistant *S. aureus* (MRSA), *E. coli*, *P. aeruginosa*, and lytic *Pseudomonas* phage)	(179)
Evening Primrose Oil	Anti-inflammatory	Human (RCTs on Rheumatoid Arthritis, Type 2 Diabetes, inflammatory conditions); *In vivo* (Rats - Adjuvant-induced arthritis model); *In vitro* (Human Endometrial Stromal Cells (HESC), Macrophages)	(180)
	Dermatological/Skin Health	Human (RCTs on Atopic Dermatitis/Eczema, improving skin hydration and barrier function); *In vivo* (Hairless Mice - UVB-irradiated photoaged skin model)	(181)
	Antioxidant	Human (Clinical Trials on diabetic patients, measuring MDA and other markers); *In vitro* (Radical scavenging assays); *In vivo* (Rats - Diabetic models)	(180)
	Metabolic/Antidiabetic	Human (RCTs on Type 2 Diabetes, Gestational Diabetes, measuring HbA_1c_ and lipid profiles); *In vivo* (Rats - Diabetic models, assessing pancreatic architecture)	(182)
	Gynecological/Women's Health	Human (RCTs on Premenstrual Syndrome (PMS), cyclical mastalgia, menopausal hot flashes); *In vitro* (Ishikawa cells, MCF-7 cells for hormonal modulation)	(182)
Grape seed oil	Anti-inflammatory	*In vivo* (Mice - Carrageenan-induced paw edema model, DSS-induced colitis; Rats - models of arthritis, allergic asthma); *In vitro* (RAW264.7 macrophages stimulated with LPS, HUVEC endothelial cells, human primary monocytes)	(183)
	Antioxidant	*In vitro* (DPPH, ABTS assays); *In vivo* (Rats - diabetic models, measuring MDA/GSH levels); Human (Clinical trials)	(184)
	Antidiabetic/Glycemic Control	*In vivo* (Rats - Diabetic models, assessing fasting blood sugar and HbA-1c); *In vitro* (Pancreatic β-cells)	(185)
	Antimicrobial	*In vitro* (Against foodborne bacteria like *L. monocytogenes*, *S. aureus*); *In vivo* (Wound healing models)	(185)
Milk thistle Oil	Antioxidant	*In vitro* (DPPH, ABTS, Hydroxyl radical scavenging assays, measuring lipid peroxidation inhibition); *In vivo* (Rats/Mice - models measuring MDA, GSH, SOD levels in liver/plasma)	(186)
	Anti-inflammatory	*In vivo* (Mice - High-Fat Diet (HFD) induced obesity/metabolic syndrome model, assessing hepatic inflammation markers like IL-6, pP65); *In vitro* (LPS-activated macrophages, human airway epithelial cells)	(187)
	Antidiabetic/Metabolic	Human (Clinical trials on Type 2 Diabetes patients, measuring glycemic indices); *In vivo* (Mice - HFD-induced obesity/hyperglycemia model)	(188)
	Antimicrobial	*In vitro* (Against Gram-positive (*S. aureus*), Gram-negative (*E. coli*), and antifungal (*C. albicans*))	(189)
Niger Oil	Anti-inflammatory	*In vivo* (Traditional/Folk use for rheumatism and inflammation; implied by content of and -tocopherol); *In vitro* (Mentioned as a general property of the seed)	(190)
	Antioxidant	*In vitro* (DPPH, ABTS assays, to measure radical scavenging activity); In Situ (Oxidative Stability Index () and shelf-life assessment of the oil)	(190)
	Antimicrobial/Antifungal	*In vitro* (Reported as a general property of the seed extract against microbes)	(190)
Orange Oil	Antimicrobial (Antibacterial/Antifungal)	*In vitro* (Micro-dilution, Disc-diffusion against *S. aureus*, *E. coli*, *C. albicans*, *L. monocytogenes*, etc.); In Situ (Food models like minced beef or bread, assessing preservative effect)	(191)
	Antioxidant	*In vitro* (DPPH, radical scavenging assays); *In vivo* (Mice - *E. coli* infected model, measuring/levels)	(192)
	Anticancer/Cytotoxicity	*In vitro* (Human cancer cell lines: leukemia cells, human colon cancer cells); *In vivo* (Rodent Models - suppressing pre-neoplastic hepatic lesions induced by DEN)	(191)
	Anti-inflammatory	*In vivo* (Implied by reduction in inflammatory markers in some human trials and established property of key monoterpenes)	(192)
Raspberry Oil	Antioxidant	*In vitro* (DPPH, ABTS, FRAP assays to measure radical scavenging activity); *In vivo* (Rats - models of oxidative stress, e.g., or -induced)	(193)
	Anti-inflammatory	*In vitro* (Macrophage cells stimulated with LPS, measuring and pro-inflammatory mediators); *In vivo* (Rats - models of induced inflammation)	(194)
	Anticancer/Cytotoxicity	*In vitro* (Against various human cancer cell lines, typically using the phenolic/ellagitannin-rich extract rather than the oil alone)	(193)
Rice Bran Oil	Antioxidant	*In vitro* (, radical scavenging assays, β-carotene/linoleate model system); *In vivo* (Albino Rats - models of oxidative stress, measuring lipid peroxidation (LPO), SOD, and CAT activity)	(195)
	Antidiabetic/Glycemic Control	Human (RCTs on patients with Type 2 Diabetes, measuring FBS, HbA_1c_); *In vivo* (Rats - /Nicotinamide-induced Type 2 Diabetes models)	(196)
	Anti-inflammatory	*In vivo* (mice, measuring inflammation and oxidative stress markers in the aorta); *In vitro* (Cell culture models - general anti-inflammatory properties of its components like -oryzanol)	(196)
	Neuroprotective	*In vivo* (General property attributed to -oryzanol and tocotrienols in animal models of neurological conditions)	(197)
Rosehip Oil	Anti-inflammatory	*In vivo* and *In vitro* assay using Inhibition of COX-1, COX-2, LTB4 enzymes, Macrophage polarization	(198)
	Antioxidant	*In vitro* assays (DPPH radical scavenging, ABTS radical scavenging, FRAP assay)	(199)
	Anti-aging	Human clinical trial (Topical application), *In vitro* (Collagen synthesis, Anti-melanogenesis in B16 mouse melanoma cells)	(200)
	Wound Healing	Human clinical trial (post-surgical scars, Second-degree burns), *In vivo* (Animal models)	(201)
Rosemary	Antioxidant	*In vitro* chemical assays using DPPH (2,2-diphenyl-1-picrylhydrazyl) and galvinoxyl free radicals.	(202)
	Antimicrobial	*In vitro* against food-borne bacteria (e.g., *E. coli*, *S. enteritidis*, *L. monocytogenes*).	(202)
	Antifungal	*In vitro* against dermatophyte fungi (e.g., *T. rubrum*, *T. mentagrophytes*).	(202)
	Hepatoprotective	*In vivo*: CCl4-induced liver injury in rats. Assessed by reduction in liver enzymes (AST, ALT, ALP) and improved histopathology.	(202)
Safflower Oil	Antidiabetic/Glycemic Control	Human (RCTs on post-menopausal obese women with Type 2 Diabetes, measuring, fasting glucose, insulin sensitivity)	(203)
	Antioxidant	*In Vitro* (Determining shelf-life stability using Peroxide, assays); In Vivo (Attributed to tocopherols, acting as radical scavengers)	(204)
	Anti-inflammatory	Human (RCTs on post-menopausal obese women with Type 2 Diabetes, measuring and levels)	(203)
	Body Composition/Anti-obesity	*In Vivo* (Male Wistar Rats - with exercise training, measuring abdominal adiposity); Human (Clinical trials using Conjugated Linoleic Acid () enriched Safflower oil)	(205)
Thyme Oil	Antimicrobial (Antibacterial/Antifungal/Antiviral)	*In Vitro* (Against food-related bacteria like *L. plantarum*, *S. aureus*, *E. coli*, and fungus like *C. albicans*); In Situ (Biofilm disruption, use in protective textiles/coatings)	(206)
	Anti-inflammatory	*In Vivo* (Mice - Carrageenan-induced pleurisy model, ear edema model, assessing inflammatory edema and leukocyte migration)	(207)
	Antioxidant	*In Vitro* (, Superoxide anion, Hydroxyl radical scavenging assays; Chinese hamster lung fibroblast cells); In Vivo (Implied by components like thymol and carvacrol)	(208)
	Antidiabetic/Hypoglycemic	*In Vivo* (Rabbits - Alloxan-induced hyperglycemic models); *In Vitro* (-glucosidase inhibition assay, typically using extracts)	(209)

#### 3.9.2 Anti-inflammatory and cardioprotective effects

Cold-pressed oils possess impressive anti-inflammatory and cardioprotective properties mainly attributed to their large number of bioactive compounds (such as MUFAs, polyphenols, and tocopherols) (213, 216, 223, 224). One such example is cold-pressed olive oil, which contains oleocanthal, a natural anti-inflammatory compound that acts like ibuprofen on inflammatory pathways, thereby decreasing chronic, low-grade inflammation associated with arthritis and cardiovascular diseases (68). Experimental studies of cold-pressed seed oils from apricot, peach, cherry, and plum demonstrated robust *in vitro* inhibition of protein denaturation, a marker of anti-inflammatory activity, that beat even standard drugs like diclofenac (57). There is robust evidence suggesting that cold-pressed oils can help improve lipid profiles in the heart, reduce the ratio of LDL to HDL cholesterol, and thereby reduce risk factors for heart disease. Population-based evidence suggests a 15–28% reduction in cardiovascular events associated with the consumption of cold-pressed oils, particularly olive oil, among those following the Mediterranean diet. These anti-inflammatory and cardioprotective actions combine to confirm the functional properties of cold-pressed oils as promising health-promoting foods in addition to being essential nutrients. Previously, several reports also studied the anti-inflammatory potential of olive oil (69), coconut oil (70), Pumpkin seed oil (66), Avocado oil (71), Mustard oil (72) and some other cold-pressed oils (Table 4). Similarly, researchers also reported the cardioprotective activity of Flaxseed (73), sunflower oil (64), Pumpkin seed oil (74, 75), and some other cold-pressed oils (Table 4). However, most of these findings are derived from preclinical studies, and their clinical relevance remains to be confirmed through well-designed human trials.

#### 3.9.3 Antimicrobial and dermatological properties

Cold-pressed oils are recognized for their potent and high-intensity antimicrobial and dermatological properties, which are attributed to their numerous bioactive compounds, including phenolics, tocopherols, and essential fatty acids. These substances enable the oils to inhibit the proliferation of various pathogenic bacteria and fungi, making them suitable for food preservation and topical use. It has been observed that cold-pressed citrus peel oils, especially from *Citrus paradisi* and *Citrus grandis*, exhibit excellent inhibitory activities against gram-positive organisms such as *Staphylococcus aureus* and *Streptococcus faecalis*, with moderate to potent (at times higher than orthodox antibiotics in inhibition zones) as well as mild against gram-negative bacteria, including *Escherichia coli* and *Salmonella enterica* (76). When oil components are hydrophobic, they inhibit microbial cell membranes through an antimicrobial action. This occurs because the hydrophobic composition of the cell membranes leads to increased permeability, allowing for higher cell contents in the resulting microbial skin membrane. Cold-pressed oils are dermatologically beneficial for skin health, offering a moisturizing effect, anti-inflammatory properties, and a strengthened skin barrier, which contributes to wound healing and reduces the risk of skin infections. Oils such as cold-pressed olive and coconut are among the widely recognized agents for calming irritated skin and combating microbial colonization, which has been established as a natural treatment in topical preparations for many years (63). Table 4 describes the antimicrobial and dermatological properties of some selected cold-pressed oils. Though, most of the available evidence comes from preclinical studies, and its applicability to humans requires confirmation through rigorous clinical research.

#### 3.9.4 Anticancer and cytoprotective potential

Oils used as hot oils have been reported to show anticancer and cytoprotection activity, not least due to their high phytochemical content of polyphenols, tocopherols, and unsaturated fatty acids. Some cold-pressed oils, such as virgin coconut oil and certain seed oils, have been demonstrated to inhibit proliferation in cell lines undergoing apoptosis in various cancers, including liver cancer, oral cancer, lung cancer, and prostate cancer, by modifying molecular pathways related to cellular cycle arrest and oxidative stress reduction (63, 77). The cytotoxic effect of these oils, which particularly include lauric acid in coconut oil and limonene in citrus seed oils, on malignant cells is supported by their low toxicity against normal cells, demonstrating their cytoprotective properties. In line with this, these oils also enhance antioxidant defense, reducing lipid peroxidation and DNA damage, and act as a dual mechanism against cancer. Therefore, cold-pressed oils provide a rich source of natural chemopreventive agents that may serve as a therapeutic adjunct to conventional therapies, warranting further clinical and mechanistic inquiries. The anticancer and cytoprotective potential of some important cold-pressed oils are listed in Table 4. However, these findings are primarily based on preclinical studies and require further clinical validation.

#### 3.9.5 Neuroprotective effects

Cold-pressed oils appear promising for neuroprotection, largely due to their high antioxidant content and bioactive compounds, including polyphenols, lignans, and monoterpenes. Cold-pressed black pepper oil, for instance, contains sesamin, piperine, and β-caryophyllene, all of which have been shown to prevent oxidative stress and reduce the amnesia observed in animal experiments by increasing the activity of antioxidant enzymes and decreasing the level of acetylcholinesterase associated with neurodegeneration. Likewise, cardamom oil has been shown to prevent the assembly of amyloid beta plaques while simultaneously upregulating brain-derived neurotrophic factor (BDNF), suggesting an important role in the management of Alzheimer’s disease. Polyphenols of olive oil (oleuropein and oleocanthal) act as neuroprotective agents by inhibiting oxidative damage and regulating anti-inflammatory pathways, helping to explain cognitive improvement observed in individuals who eat the Mediterranean diet (78). Additionally, virgin coconut oil exhibits neuroprotective properties by alleviating oxidative stress and inflammation in models of neurodegeneration (70). These findings suggest that cold-pressed oils may be a valuable adjunct in preventing and slowing neurodegenerative diseases due to their diverse protective actions in neurodegeneration through a well-balanced mechanism of action. Researchers also studied the neuroprotective activity of groundnut oil (79). However, these results are primarily grounded on preclinical studies and require additional clinical validation.

#### 3.9.6 Antidiabetic effects

Cold-pressed oils have also demonstrated strong antidiabetic effects through several mechanisms, including enhanced insulin sensitivity, decreased blood glucose levels, and modification of the lipid profile. Virgin coconut oil, rich in medium-chain triglycerides (MCTs) and lauric acid, has previously been shown to increase insulin sensitivity by up to 40%, reduce fasting blood glucose levels, and decrease the postprandial hyperglycemic phase in diabetic subjects, among other benefits (52). Likewise, oils such as sesame and flaxseed contain bioactive substances, namely lignans and omega-3 fatty acids. These compounds exert anti-inflammatory effects and improve glycemic control (80). Several cold-pressed oils, as the study authors concluded, function as helpful adjuncts in diabetes control, enhancing metabolic health and preventing secondary diabetes complications; however, further testing in healthy humans is still required for these findings to be validated. The details of the antidiabetic effects of some important cold-pressed oils are shown in Table 4. However, most of these findings are derived from preclinical studies, and their clinical relevance remains to be confirmed through well-designed human trials.

#### 3.9.7 Hepatoprotective activity

The cold-pressed oils exhibited remarkable hepatoprotective activity, owing to their rich content in antioxidants, polyunsaturated fatty acids, and bioactive compounds, including tocopherols and phenolics (217, 223). In experimental analyses, it has been demonstrated that cold-pressed oils, such as clove oil and virgin coconut oil, mitigate chemically induced liver injuries by inhibiting oxidative stress indicators, including malondialdehyde (MDA), and increasing antioxidant catalysts, such as glutathione (GSH), in hepatic tissues. These oils have been reported to lower serum liver enzyme activities (AST, ALT, ALP), lipids, and inflammatory markers, thereby protecting liver cells from necrosis, fatty degeneration, and fibrosis. Such findings reinforce the therapeutic potential of cold-pressed oils as natural agents for preventing or counteracting oxidative liver disorders (81), and also demonstrate how cold-pressed oils can redirect the oxidative process. Although, most of these reports are derived from preclinical studies, and their clinical significance remains to be established through well-designed human trials.

## 4 Discussion

The present systematic review synthesizes physicochemical, nutritional, bioactive, and pharmacological data from 216 studies on cold-pressed edible oils. The collective evidence demonstrates that cold pressing preserves a wide spectrum of beneficial lipids, antioxidants, and micronutrients that are significantly diminished during conventional refining processes. This discussion interprets the findings in the context of current scientific understanding, comparing oils across categories, identifying major biochemical and functional trends, and highlighting implications for nutrition, food science, and therapeutic research.

### 4.1 Physicochemical integrity and its implications for oil quality

Cold-pressed oils consistently demonstrate favorable physicochemical properties, confirming the benefits of mechanical extraction without the use of heat or solvents. Low free fatty acid (FFA) levels typically below 1% in oils such as olive, avocado, almond, and groundnut indicate minimal hydrolytic degradation and effective seed handling (28, 29). Slightly higher FFAs in cumin, coriander, and cinnamon oils reflect intrinsic enzymatic activity rather than processing flaws. Even highly polyunsaturated oils, including flaxseed and hempseed, maintained acceptable FFAs when seeds were properly dried and stored, showing that cold pressing is suitable for PUFA-rich matrices.

Peroxide values (PVs) remained within acceptable quality limits across studies, demonstrating that cold pressing does not promote oxidative stress despite the vulnerability of PUFA-rich oils (26). This stability is aided by endogenous antioxidants tocopherols, phenolics, and sterols that are preserved during cold extraction. Iodine values (IVs) varied predictably with unsaturation: PUFA-rich oils displayed high IVs, MUFA-dominant oils moderate IVs, and saturated oils low IVs, reflecting a balance between oxidative stability and nutritional benefit (82). Additional parameters, including saponification value, specific gravity, and unsaponifiable matter, highlighted the biochemical richness of cold-pressed oils. High unsaponifiable fractions in sesame, olive, avocado, and argan oils underscore their superior content of bioactive compounds that contribute to antioxidant, cardioprotective, and immune-supporting health effects.

A comparative evaluation of key physicochemical parameters (FFA, PV, IV, and SV) across studies reveals clear quantitative trends that are strongly dependent on oil source. Oils rich in polyunsaturated fatty acids, such as flaxseed and chia, consistently exhibit higher peroxide and iodine values, reflecting increased unsaturation and susceptibility to oxidation, whereas saturated and monounsaturated oils, including coconut and olive, show lower peroxide values and greater oxidative stability. Variations in free fatty acid content are also influenced by intrinsic seed properties, such as enzymatic activity and microstructure, rather than processing alone. Similarly, differences in saponification values correspond to fatty acid chain length distribution inherent to specific oil types. These observations indicate that the botanical origin and compositional profile of the oil are primary determinants of physicochemical behavior, while processing conditions play a secondary modifying role (39).

### 4.2 Nutritional composition: diversity and functional significance

Cold-pressed oils exhibit diverse fatty acid profiles, enabling targeted nutritional applications. Saturated fatty acid rich oils like coconut and palm show high oxidative stability, with coconut oil’s medium-chain SFAs supporting rapid energy use and antimicrobial effects. MUFA-rich oils such as olive, avocado, almond, and argan promote cardiometabolic health through oleic acid-mediated improvements in lipid balance, inflammation, and oxidative stress (4, 43). PUFA-rich oils including flaxseed, chia, hempseed, sunflower, walnut, and pomegranate provide essential fatty acids crucial for neurodevelopment, immunity, and metabolic regulation (39). Cold pressing preserves these sensitive PUFAs, enhancing the functional benefits of oils like ALA-rich flaxseed and anti-inflammatory punicic acid rich pomegranate oil (2).

### 4.3 Bioactive composition: phenolics, tocopherols, sterols, and terpenes

This review shows that cold pressing preserves significantly higher levels of bioactive compounds than refining, enhancing antioxidant, anti-inflammatory, and metabolic benefits. Phenolic-rich oils like olive, sesame, argan, black seed, and pomegranate deliver strong radical-scavenging, cardioprotective, and lipid-modulating effects (1, 7). Tocopherols especially α-, γ-, and δ-forms remain intact due to minimal heat exposure, supporting antioxidant defense, neurological health, and immunity (1). Terpenes, triterpenoids, and sterols in oils such as olive, argan, sesame, and mustard contribute to oxidative stability and cholesterol reduction. Unique bioactives like thymoquinone, punicic acid, glucosinolates, and MCTs highlight each oil’s distinct functional and therapeutic potential.

### 4.4 Pharmacological properties and therapeutic potential

While numerous studies report promising health and pharmacological effects of cold-pressed oils, it is important to interpret these findings cautiously in light of the evidence level. A substantial proportion of the reported antioxidant, anti-inflammatory, and anticancer activities are derived from *in vitro* and animal studies, with comparatively limited clinical validation in humans (1, 63). Therefore, these effects should be considered indicative rather than conclusive. To ensure balanced interpretation, the present review integrates findings from multiple studies and emphasizes consistency of outcomes rather than isolated results. Future well-designed clinical trials are required to substantiate these bioactivities and establish their translational relevance. Cold-pressed oils exhibited diverse pharmacological effects, driven by their preserved fatty acids and rich bioactive compounds. The pharmacological effects discussed are largely attributed to preserved bioactive constituents resulting from cold-press extraction, rather than being direct clinical outcomes of cold-pressed oils per se, and are primarily supported by *in vitro* and animal studies with limited clinical validation. Strong antioxidant and anti-inflammatory activities were noted in sesame, olive, black seed, pumpkin seed, and pomegranate oils, with compounds like oleocanthal and thymoquinone targeting COX and NF-κB pathways. MUFA- and PUFA-rich oils improved lipid profiles, vascular function, oxidative stress, and insulin sensitivity, with ALA-rich flaxseed, chia, and hempseed showing potent cardiometabolic benefits (12, 68). Antimicrobial activity was pronounced in coconut, mustard, black seed, and pomegranate oils (63). Organ-protective properties hepatic, renal, and neurological were evident in pumpkin seed, olive, pomegranate, and black seed oils (83).

The pharmacological effects described in this review should be interpreted with caution, as the majority of evidence is derived from *in vitro* experiments and animal models rather than well-controlled human clinical trials. While cold-press extraction preserves bioactive compounds that are known to exhibit biological activity, direct clinical evidence linking cold-pressed oils to disease prevention or therapeutic outcomes remains limited. Therefore, these findings should not be considered as clinically established effects but rather as indicative of potential biological activity. Future research should focus on well-designed human studies to validate these observations and establish clear cause-effect relationships.

### 4.5 Integrative interpretation and practical implications

Cold-pressed oils represent a diverse and biochemically rich category of functional foods. Their unique compositions make them suitable for specific nutritional, culinary, and therapeutic applications:

- High-PUFA oils (flaxseed, hempseed, pomegranate) → best for raw consumption and therapeutic supplementation
- High-MUFA oils (olive, avocado, almond, argan) → suitable for both raw and light cooking applications
- High-SFA oils (coconut, palm kernel) → ideal for high-temperature cooking and antimicrobial uses
- Phenolic-rich oils (olive, sesame, black seed) → strong candidates for anti-inflammatory and antioxidant therapies

This systematic review underscores the importance of preserving the natural composition of oils through cold pressing. The synergistic interactions among fatty acids, polyphenols, tocopherols, sterols, and terpenes contribute to the unique nutritional and pharmacological attributes of each oil, supporting their role in dietary intervention strategies and natural product-based therapeutics.

### 4.6 Challenges and limitations

Despite the numerous advantages, cold-pressed oils also face several challenges, particularly in terms of stability and shelf life. One of the primary issues is the relatively short shelf life of these oils, especially those rich in polyunsaturated fatty acids (PUFAs), such as flaxseed, sunflower, and hempseed oils. PUFAs are highly susceptible to oxidation, which leads to rancidity and the loss of flavor, aroma, and nutritional value. This oxidation process is accelerated by exposure to light, heat, and oxygen, making proper storage critical to preserving the quality of cold-pressed oils. While refining processes can increase shelf life by removing reactive compounds, this benefit comes at the cost of nutrient loss. To address the oxidation problem in cold-pressed oils, several methods have been proposed. These include fortifying oils with natural antioxidants like vitamin E, rosemary extract, and ascorbic palmitate, which can help delay oxidation and improve shelf stability. Additionally, controlled storage techniques, such as storing oils in dark glass bottles, maintaining them in cool environments, and utilizing modified atmosphere packaging (MAP), are crucial in extending their shelf life. However, the inherent instability of cold-pressed oils remains a significant challenge that requires continuous attention from both researchers and producers to develop more effective preservation strategies. The review also found that natural antioxidants preserved through cold pressing significantly improved shelf life, suggesting that the oxidative vulnerability of PUFA-rich oils can be mitigated by high phenolic and tocopherol content when seeds are properly stored before extraction.

### 4.7 Research gaps and future directions

While considerable progress has been made in understanding the health benefits and nutritional properties of cold-pressed oils, several research gaps remain that need to be addressed. One of the key areas of focus is the oxidative stability of cold-pressed oils, particularly those rich in PUFAs. Although some methods to improve oxidative stability have been explored, such as antioxidant fortification and packaging innovations, there remains a need for more comprehensive studies on how to best preserve the bioactive components of these oils over time. Furthermore, the role of cold-pressed oils in the prevention and management of chronic diseases, such as cancer, cardiovascular disease, and metabolic disorders, warrants further investigation. Numerous studies have demonstrated the health benefits of individual cold-pressed oils; however, more clinical trials are needed to validate their long-term efficacy and safety in disease prevention and management. Additionally, research should focus on understanding the impact of different pre-processing and extraction methods on the bioactive value of oils. Factors such as seed moisture content, extraction temperature, and pressure can all affect the final composition of the oil and, consequently, its therapeutic potential. Addressing these gaps will enable a more comprehensive understanding of how cold-pressed oils can be optimized for their health benefits and their role in promoting sustainable, health-conscious food systems.

### 4.8 Speculative theories

Future research could explore the synergistic effects of combining cold-pressed oils with other nutraceuticals, such as polyphenol-rich extracts, to enhance their therapeutic potential. The incorporation of natural compounds, such as flavonoids, curcumin, or green tea extracts, into cold-pressed oils may create new functional food products with enhanced antioxidant, anti-inflammatory, and anticancer properties. Additionally, advancements in oil packaging and preservation technologies are crucial for addressing the stability issues associated with cold-pressed oils. The development of innovative packaging materials, such as oxygen-absorbing or light-blocking containers, and the use of microencapsulation or nanotechnology to protect sensitive bioactive compounds from oxidation could significantly improve the shelf life of cold-pressed oils without compromising their nutritional integrity. These speculative theories suggest that there is considerable potential for innovation in the cold-pressed oil industry, particularly in areas such as product formulation and packaging, which could lead to more stable, shelf-stable oils that retain their health benefits over time.

## 5 Conclusion

This systematic review demonstrates that cold-pressed edible oils constitute a uniquely valuable category of functional foods, distinguished by their preserved fatty acid profiles, high concentrations of bioactive compounds, and multifaceted health-promoting properties. By avoiding the high heat and chemical solvents used in conventional refining, cold pressing maintains the natural biochemical integrity of oils including essential PUFAs and MUFAs, tocopherols, polyphenols, phytosterols, terpenes, and unique seed-derived metabolites. These components collectively contribute to superior antioxidant capacity, enhanced anti-inflammatory effects, and a wide range of therapeutic benefits, including cardioprotective, hepatoprotective, antimicrobial, neuroprotective, and metabolic actions.

The findings also reveal substantial inter-oil variability, underscoring that cold-pressed oils are not interchangeable but rather possess distinct compositional and pharmacological signatures. PUFA-rich oils (flaxseed, chia, hemp, pomegranate) offer potent bioactivity but require careful storage due to oxidation susceptibility, whereas MUFA-rich oils (olive, avocado, almond, argan) provide an optimal balance of stability and health benefits. SFA-rich oils (coconut, palm kernel) contribute valuable antimicrobial and metabolic functions. Phenolic- and lignan-rich oils (sesame, olive, black seed) demonstrate exceptional therapeutic potential.

Collectively, the evidence supports the integration of cold-pressed oils into dietary and therapeutic frameworks, including functional foods, nutraceuticals, dietary supplementation, and targeted clinical applications. Future research should explore standardized extraction conditions, bioavailability of oil constituents, synergistic interactions among phytochemicals, and the development of stable formulations for PUFA-rich oils. This review highlights cold-pressed oils as promising candidates for advancing nutrition science, chronic disease prevention, and natural health product development.

## Data Availability

The original contributions presented in the study are included in the article/supplementary material, further inquiries can be directed to the corresponding author/s.
